# Differential impacts of late gestational over–and undernutrition on adipose tissue traits and associated visceral obesity risk upon exposure to a postnatal high‐fat diet in adolescent sheep

**DOI:** 10.14814/phy2.14359

**Published:** 2020-02-05

**Authors:** Prabhat Khanal, Deepak Pandey, Sharmila Binti Ahmad, Sina Safayi, Haja N. Kadarmideen, Mette Olaf Nielsen

**Affiliations:** ^1^ Animal Science, Production and Welfare Division Faculty of Biosciences and Aquaculture Nord University Steinkjer Campus Norway; ^2^ Department of Veterinary and Animal Sciences Faculty of Health and Medical Sciences University of Copenhagen, Denmark Frederiksberg Denmark; ^3^ Graduate College Rush University Chicago IL USA; ^4^ Department of Applied Mathematics and Computer Science Technical University of Denmark Kongens Lyngby Denmark; ^5^ Department of Animal Science Aarhus University Tjele Denmark

**Keywords:** adipocyte tissue, cellularity, fetal programming, hyperplasia, hypertrophy

## Abstract

We hypothesized that late gestation malnutrition differentially affects expandability of adipose tissues to predispose for early postnatal visceral adiposity. Twin‐lambs born to dams fed HIGH (150%/110% of required energy/protein, respectively), NORM (100% of requirements) or LOW (50% of NORM) diets during the last trimester were used. Postnatally, lambs were raised on moderate (CONV) or high‐carbohydrate‐high‐fat (HCHF) diets. Adipose tissues were sampled at autopsy at 6 months of age (~puberty) to characterize cellularity, adipocyte cross‐sectional area and gene expression patterns. HIGH and LOW compared to NORM lambs had reduced intrinsic (under CONV diet) cellularity in subcutaneous and mesenteric (particularly LOW), and reduced obesity‐induced (under HCHF diet) hyperplasia in subcutaneous, mesenteric and perirenal (particularly HIGH) adipose tissues. This corresponded with more pronounced HCHF diet‐induced hypertrophy in mesenteric (particularly LOW), perirenal (particularly HIGH) and subcutaneous (particularly HIGH) adipose tissues, and tissue‐specific reductions in mRNA expressions for lipid metabolism, angiogenesis and adipose development. Gene expression for inflammation and lipid metabolism markers were increased and decreased, respectively, in HCHF lambs (HCHF lambs became obese) in all tissues. Both prenatal over‐ and undernutrition predisposed for abdominal adiposity and extreme perirenal hypertrophy due to reduced intrinsic (observed under CONV diet) cellularity and impaired ability of subcutaneous, mesenteric and perirenal adipose tissues to expand by hyperplasia rather than hypertrophy on an obesogenic (HCHF) diet.

## Graphical abstract

Both prenatal over‐ and undernutrition predisposes for abdominal adiposity and extreme perirenal hypertrophy due to reduced intrinsic cellularity and impaired ability of subcutaneous, mesenteric and perirenal adipose tissues to expand by hyperplasia rather than hypertrophy.

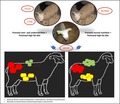

## 1 INTRODUCTION

It is now well accepted that maternal nutrient restriction during gestation, followed by a mismatching subsequent overnutrition in early life, is associated with increased obesity risk (Cleal et al., 2007; Gluckman, Hanson, & Spencer, 2005; Yan et al., 2017). In recent years, it has become recognized that adipose tissue must be one of the major targets of the so‐called fetal programming (Symonds, Pope, Sharkey, & Budge, 2012) linking prenatal undernutrition to increased prevalence of obesity in postnatal life. In this way, the contribution of adverse fetal nutrition to the present global increase in prevalence of obesity (Desai & Ross., 2011) must be acknowledged.

Although studies on intrauterine overnutrition are scarce, some evidence do exist suggesting that maternal overnutrition can have similar adverse impacts as maternal undernutrition on adiposity risk and fat deposition patterns later in life. Thus, previous studies in higher animals like sheep and pigs have shown that dietary interventions in the form of gestational over‐ (Giblin et al., 2015; Long, Rule, Tuersunjiang, Nathanielsz, & Ford, 2015; Muhlhausler, Duffield, & McMillen, 2007a, 2007b) or undernutrition (Ford et al., 2007; Yan et al., 2017) similarly can give rise to increased fat deposition and visceral adiposity in the offspring. In sheep studies conducted by our group, we have in agreement with these observations found that maternal malnutrition both in the form of over‐ or undernutrition during late gestation results in altered fat deposition patterns, resulting in a higher visceral to subcutaneous fat ratio in the offspring (Khanal et al., 2014; Nielsen et al., 2013). The precise mechanisms underlying such altered fat distribution patterns are not known, but it has been proposed to be associated with a reduced expandability of subcutaneous adipose tissue, which can lead to increased visceral adipose tissue mass upon exposure to an obesogenic diet to a nutrient overflow situation (Sniderman, Bhopal, Prabhakaran, Sarrafzadegan, & Tchernof, 2007). In fact, subcutaneous fat is considered as a “healthy” fat depot, since it can act as a nutrient “sink” in situations of excessive energy availability, and it also has unique intrinsic properties to provide beneficial impacts on whole‐body metabolism (Tran, Yamamoto, Gesta, & Kahn, 2008). Thus, poor expandability of subcutaneous fat may increase the risk of nutrient overflow and excess accumulation of abdominal fat (Spalding et al., 2008, 2017).

Previous studies have suggested that alterations in maternal nutrient supply during pregnancy can lead to changes in expression patterns of genes associated with lipid metabolism and inflammatory responses in adipose tissues of offspring. Maternal protein restriction throughout the gestation and lactation period upregulated the expressions of the glucose transporter‐4 (GLUT4) gene, a key element for glucose uptake, as well as fatty acid synthase (FAS) and CCAAT/enhancer‐binding protein (C/EBP)‐β genes in visceral adipose tissue in rat offspring (Guan et al., 2005). Other studies have demonstrated that late gestational nutrient restriction in sheep leads to overexpression of inflammatory markers such as cluster of differentiation 68 (CD68) and toll‐like receptor 4 (TLR4) in perirenal adipose tissue of the offspring (Sharkey, Symonds, & Budge, 2009), and in rodents early postnatal high‐fat diet was associated with adipose tissue inflammation with up‐regulation of monocyte chemoattractant protein‐1 (MCP‐1) and CD68 (Kayser, Goran, & Bouret, 2015; Xu et al., 2003). These studies demonstrate that glucose and lipid metabolism pathways as well as inflammatory processes in adipose tissue could be possible targets of fetal programming, and the question is, whether this could contribute to explain the proposed link between fetal malnutrition and increased obesity risk later in life. Gestational overnutrition and being born large‐for‐gestational‐age are now also increasingly recognized as predisposing factors for adiposity later in life (Ojha, Saroha, Symonds, & Budge, 2013; Rajia, Chen, & Morris, 2010), but the mechanisms behind the association between maternal overnutrition and adipose tissue development and inflammation remain to be explored.

In this study, we used the Copenhagen sheep model (Khanal et al., 2014; Nielsen et al., 2013) to test the hypotheses that 1) both late gestational under‐ and overnutrition diminish the postnatal expandability of adipose tissues in a differential way, thereby predisposing for excess fat deposition in the abdominal regions upon exposure to an early postnatal high‐fat, obesogenic diet, and 2) this is associated with differential changes in cellularity in adipose tissues (subcutaneous, mesenteric, perirenal and epicardial).

## 2 MATERIALS AND METHODS

### 2.1 Experimental animals and treatments

**Figure 1:**
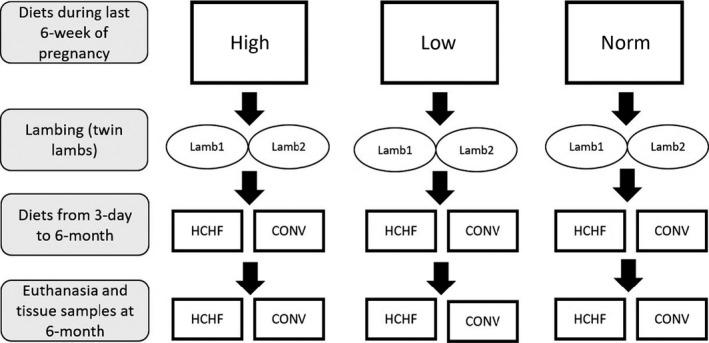
Experimental design (For details, see (Khanal et al., 2014)). HIGH: diet fed to twin‐pregnant dams during the last trimester and fulfilling 150% of their daily energy and 110% of their daily protein requirements (*N* = 10; four males, six females); LOW: diet fed to twin‐pregnant dams during the last trimester and fulfilling 50% of their daily energy and protein requirements (*N* = 10; 5 males, 5 females;); NORM: diet fed to twin‐pregnant dams during the last trimester and fulfilling 100% of their daily energy and protein requirements (*N* = 6; six males, 0 female;); HCHF: high‐carbohydrate, high‐fat postnatal diet fed to lambs and consisting of cream‐milk replacer mix in a 1:1 ratio supplemented with rolled maize (*N* = 13; 8 males, 5 females) and CONV: conventional postnatal diet fed to lambs and consisting of milk replacer and hay until 8 weeks of age and hay only thereafter and adjusted to achieve moderate and constant growth rates of approx. 225 g/day (*N* = 13; 7 males, 6 females)

All the experimental animal handling protocols were approved by the Danish National Committee on Animal Experimentation. This study was conducted using a sub‐group of animals from a larger experiment, which was conducted at the experimental facilities on the Rosenlund farm, Lynge, Denmark under the auspices of the Faculty of Health and Medical Sciences, University of Copenhagen, Denmark. The experimental animals, dietary treatments, and their management have previously been described in detail (Khanal et al., 2014) and are outlined in Figure 1. In short, the study was a 3 × 2 factorial design experiment, and for this part of the study, 26 lambs born as twins from 26 twin pregnant ewes were used. The ewes had been subjected to three different diets in late gestation: overnutrition (HIGH; where daily amounts of feed ingested corresponded to 150% and 110% of requirements for energy and protein, respectively, as specified by (NRC, 2007); *N* = 10), moderate nutrition (NORM; fulfilling requirements for energy and protein; *N* = 6), and undernutrition (LOW; fulfilling 50% of energy and protein requirements; *N* = 10). From 3 days after birth until six months of age (just after puberty), the twin lambs were assigned to either a high‐carbohydrate, high‐fat diet (HCHF; max. 2 L/d of a 1:1 milk replacer‐dairy cream mix and max. 2 kg/day of rolled maize; *N* = 13) or a moderate low‐fat conventional diet (CONV; hay supplemented with milk replacer during the first 8 weeks of life and hay only thereafter; feed allocation was adjusted weekly so that moderate growth rates of appr. 225 g/day were achieved; *N* = 13). Thus, six different treatment groups exposed to matched or mismatched nutrition pre‐ and postnatally were created: HIGH‐HCHF, HIGH‐CONV, NORM‐HCHF, NORM‐CONV, LOW‐HCHF, and LOW‐CONV.

### 2.2 Tissue sampling and processing

At 6 months of age, subgroups of animals from each treatment group were humanely euthanized in such a way that there would be as uniform a distribution of gender as possible in slaughtered and surviving animals to be able to subsequently assess the consequences in both genders as adults of different early life nutrition exposures. The exception was that only males were slaughtered from the NORM‐CONV and NORM‐HCHF groups due to a smaller group size. Therefore, when evaluating results from the present study, it should be borne in mind that gender effects could only be evaluated for the LOW and HIGH prenatal groups and not for the animals from the prenatal NORM group, as reported earlier (Khanal et al., 2014). Tissues were sampled from subcutaneous (above the *muscularis longissimus dorsii* at the level of the last rib), the epicardial (from the anterior surface of the heart), and randomly from mesenteric and perirenal adipose tissues. For gene expression studies, pieces of tissues were immediately submerged in RNA*later* (RNA*later®* Solution, Ambion, The RNA Company, USA) for 24 hr and then all samples were stored at −80°C pending analyses. For histological evaluations, adipose tissue samples were fixated in 4% paraformaldehyde (PFA) for 24 hr, and afterwards in 1% PFA for a week until paraffin embedding.

### 2.3 Histology

The PFA‐fixed tissue samples embedded into paraffin blocks were used for tissue sectioning. Five µm thick sections were cut using a Leica sliding microtome (Leica Microsystems, Ballerup, Denmark) and mounted on a superfrost glass slide (SuperFrost® WHITE; Hounsen Laboratorieudstyr, Århus, Denmark). In every tissue block, 300 µm was cut off and discarded after the first collection, and this was repeated twice yielding sections from three different collection sites within each tissue sample. To ensure unbiased results, each slide was assigned a number to mask its identity throughout the analysis process. After sectioning, tissue slides were put in a heating oven at 50°C for 40 min and thereafter stored at room temperature until staining. The slides were stained according to the protocol by van Gieson, as reported previously (Khanal et al., 2014).

**Figure 2:**
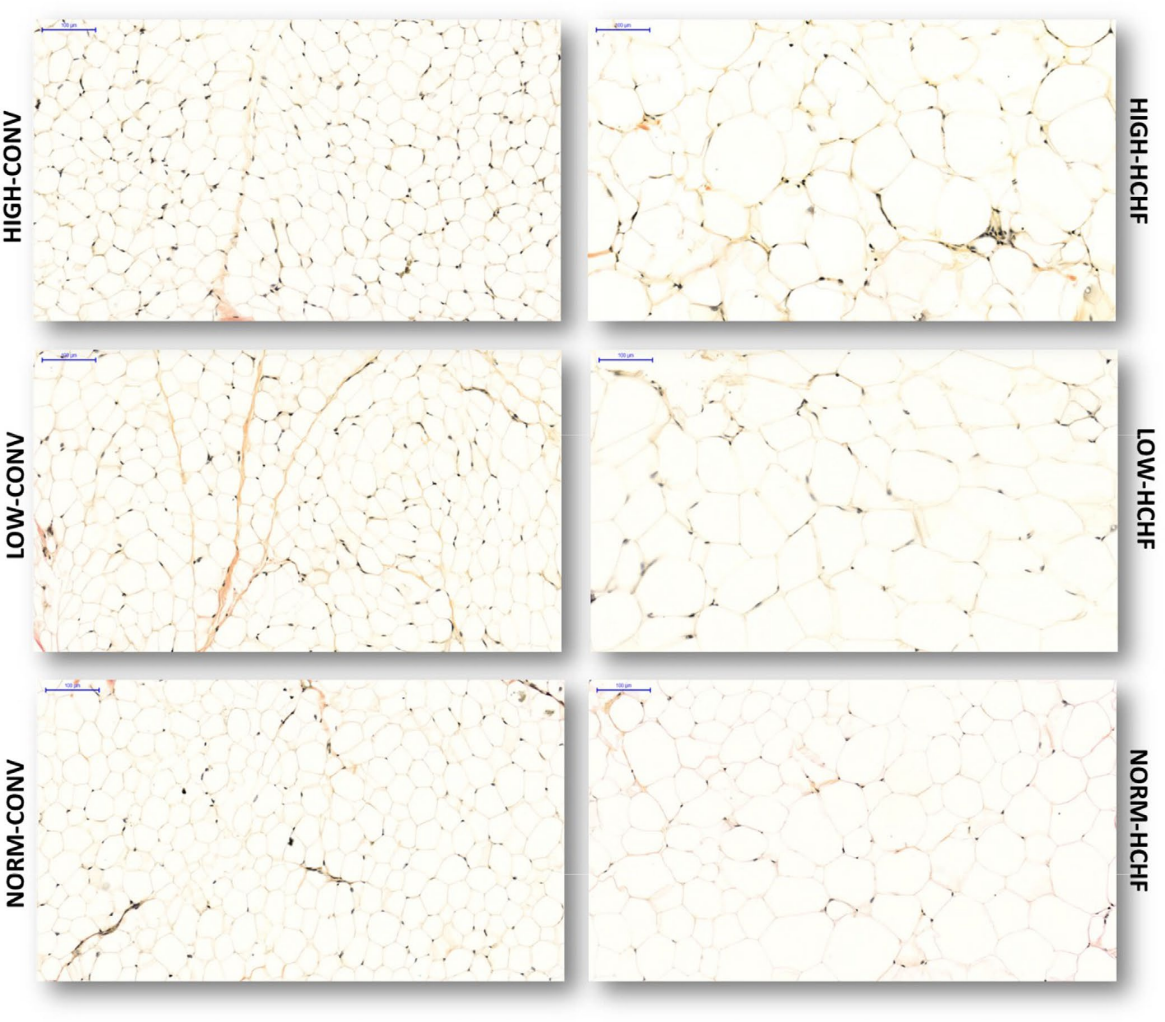
Histological evaluation of adipocytes in subcutaneous adipose tissue. Five µm thick tissue sections were stained by van Gieson staining; the tissue slides were scanned by a panoramic whole slide scanner and the pictures were taken at 20× magnification. Scale bar is 100 µm for all pictures. HIGH, LOW, NORM, HCHF, and CONV: see legend to Figure 1

**Figure 3:**
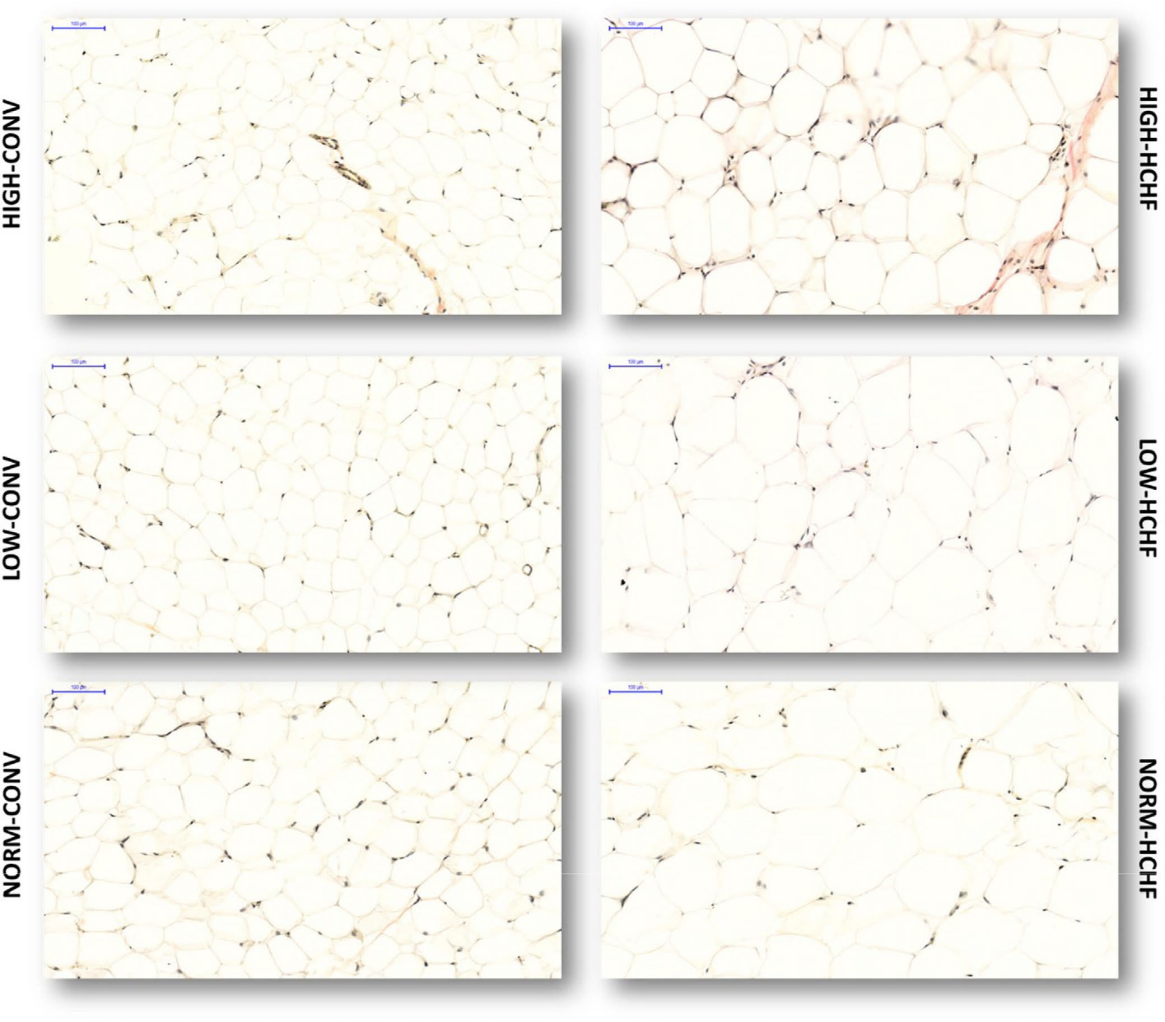
Histological evaluation of adipocytes in mesenteric adipose tissue. Five µm thick tissue sections were stained by van Gieson staining; the tissue slides were scanned by a panoramic whole slide scanner and the pictures were taken at 20× magnification. Scale bar is 100 µm for all pictures. HIGH, LOW, NORM, HCHF and CONV: see legend to Figure 1

**Figure 4:**
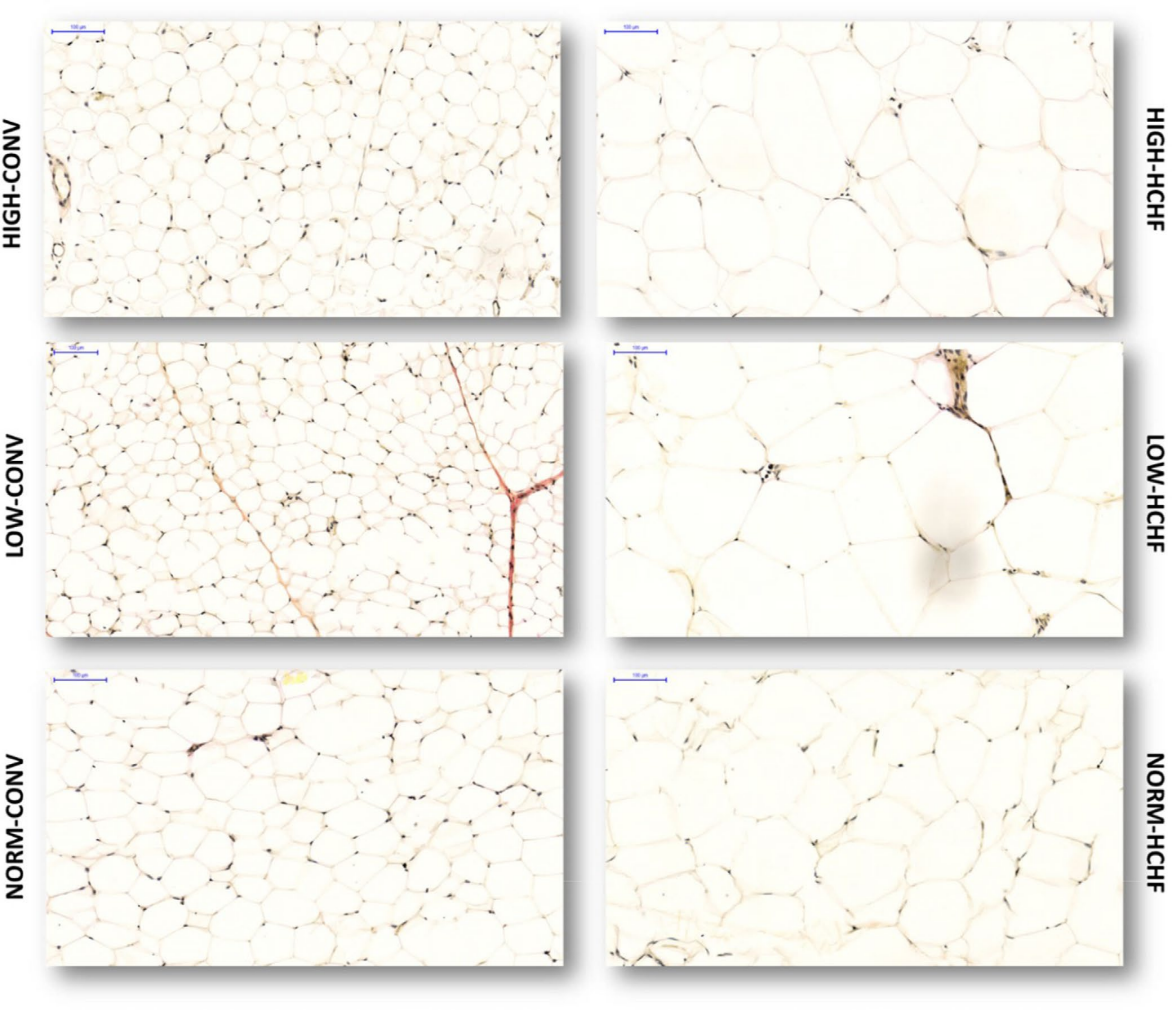
Histological evaluation of adipocytes in perirenal adipose tissue. Five µm thick tissue sections were stained by van Gieson staining; the tissue slides were scanned by a panoramic whole slide scanner and the pictures were taken at 20× magnification. Scale bar is 100 µm for all pictures. HIGH, LOW, NORM, HCHF, and CONV: see legend to Figure 1

The stained tissue slides were scanned by a Panoramic MIDI whole slide scanner (3DHISTECH Ltd, Konkoly‐Thege M. str., Budapest, Hungary) and a total of five pictures were randomly taken from each section at 20× magnification, providing a total of fifteen pictures for histology analyses from each adipose tissue depot per animal. The relative proportion of different tissue structures in the slides were evaluated using ImageJ software (Abràmoff, Magalhães, & Ram, 2004) by randomly applying a 28‐points transparent grid on each picture as described (Safayi et al., 2010). The tissue structures were classified as adipocytes, collagen fibres or microvessels, and proportions of these structures referred to the number of hits on a given cell structure relative to the total number of hits in the whole picture. The counting of coinciding points (420 hits per sample) with the above‐mentioned desired structures allowed unbiased estimation (Gundersen et al., 1988) of volume fractions for each animal. Adipocyte cross‐sectional area (CSA) was determined manually and adipocytes used for CSA measurements were chosen by randomly assigning a 15‐points transparent grid. A cell number index (CNI) was calculated for subcutaneous, mesenteric and perirenal adipose tissues as: adipocyte mass (total fat mass (kg) multiplied by the percentage of adipocytes in the tissue) divided by the cell volume of a spherical adipocyte with a radius derived from the measured mean CSA: CSA = π × *r* ^2^. The CNI is obviously not an estimate of the total number of adipocytes in the respective tissues, since adipocytes are not spherical but have an angular shape (particularly in HCHF fed lambs; see Figures 2, 3, 4), and the mean CSA determined in the slides will expectedly be lower than the actual CSA at the center of the adipocyte, since the cells counted obviously will represent cells cut at different distances relative to the center. However, the calculated CNI should allow us to evaluate, whether differences in fat deposition patterns in response to different pre‐and postnatal nutritional exposures would be a result of changes in adipocyte size (hypertrophy) or potentially also a result of changes in adipocyte numbers (hyperplasia).

### 2.4 Quantitative real‐time PCR (qPCR)

#### 2.4.1 RNA extraction and cDNA synthesis

To extract total mRNA, about 300 mg of adipose tissue was homogenized in 2000 µl TRIzol® reagent (Invitrogen, Life Technologies Europe BV) on a gentleMACS homogenizer (Miltenyi Biotec, Bergisch Gladbach) and about 600 µl supernatant solution was used for phase separation by using 120 µl 1‐Bromo‐3‐Chloropropane (Sigma‐Aldrich). Then the upper colorless aqueous phase was removed for precipitation of RNA with 500 µl of isopropanol (Sigma‐Aldrich) for 1 hr at −20°C. RNA was subsequently purified using the Promega RNA Total Isolation kit (Promega Corporation), and RNA concentrations and integrity of isolated RNAs were analysed as previously described (Hou et al., 2013). To synthesize cDNA, 0.5 µg of total RNA was used for reverse transcription, which was performed as previously described (Hou et al., 2013). All cDNA samples were stored at −20°C until further analysis.

#### 2.4.2 Quantitative real‐time PCR (qPCR)

The mRNA expression levels of target genes in different adipose tissue depots were determined by qPCR. The cDNA was pooled to make standard curves and a calibrator for each plate. Standard curves were made using serial dilutions of cDNA (1:2, 1:4, 1:8, 1:16, 1:32, and 1:64) to determine the efficiency of each primer set within the resulting linear regression. Efficiencies of primers were between 1.84 and 1.93 (this equates to an increase between 85% and 92% of target nucleic acid in each amplification cycle) (Table 1). Calibrators, samples and negative controls were performed in triplicate. The mRNA expression levels of 14 target genes (see Table 1) were determined in all the sampled adipose tissues using LightCycler® 480 SYBER Green I Master (Roche Applied Science) and LightCycler® 480 (Roche Applied Science) systems. The reaction volume of each well was 10 μl, which contained 2 μl ten‐times diluted cDNA, 5 μl 2 × SYBR Green I master mix (Roche Applied Science), 1 μl 10 μM forward primer (TAG Copenhagen, Copenhagen, Denmark), 1 μl 10 μM reverse primer (TAG Copenhagen), and 1 μl nuclease‐free water (Roche Applied Science). Samples and negative controls (no cDNA template) were run in an amplification cycle program including denaturation (95°C for 10 s), annealing (58–60°C for 10 s) and elongation (72°C for 20 s) that was repeated 45 times in each qPCR reaction. Melting curves of PCR products were analyzed by a LightCycler 480 instrument ver. 2.0 software (Roche Applied Science) to ensure that a single product was obtained, and the PCR‐product size was confirmed by agarose gel electrophoresis.

**Table 1:** Primer sequences used in q‐PCR

Gene	Forward primer	Reverse primer	Primer efficiency	Product size (bp)
FASN	5′‐CCCAGCTCAACGAAACCA−3′	5′‐GACGAGGTCAACACCCTTCC−3′	1.850	95
FABP4	5′‐CATCTTGCTGAAAGCTGCAC−3′	5′‐AGCCACTTTCCTGGTAGCAA−3′	1.846	160
GLUT4	5′‐AGTATGTGGCGGATGCTATGG G−3′	5′‐CGGCGGAAGACGGCTGAG−3′	1.918	139
HSL	5′‐GGTAATTGCCGACTTCCTACGA−3′	5′‐TGGCAGGTGTGAACTGGAAA−3′	2	89
LPL	5′‐CGGCTTTGATATTGGGAAGA−3′	5′‐CCGGGAACAGAAGATCACC−3′	2	70
PPAR‐γ	5′‐ACGGGAAAGACGACAGACAAATC−3	5′‐CACGGAGCGAAACTGACACC−3′	1.91	159
C/EBP‐β	5′‐ACGACTTCCTCTCCGACCTC−3′	5′‐CCCAGACTCACGTAGCCGTA−3′	1.918	85
VEGFA	5′‐GCCTTGCCTTGCTGCTCT/AC3′	5′‐GGTTTCTGCCCTCCTTCTGC−3′	1.860	75
GAPDH	5′‐GTCGGAGTGAACGGATTTGG−3′	5′‐AACGATGTCCACTTTGCCAGTA−3′	1.904	86
TNF‐α	5′‐AACAGGCCTCTGGTTCAGACA−3′	5′‐CCATGAGGGCATTGGCATAC−3′	1.927	136
IL−6	5′‐GACACCACCCCAAGCAGACTA−3′	5′‐TGCCAGTGTCTCCTTGCTGTT−3′	1.993	144
MCP−1	5′‐GCTGTGATTTTCAAGACCATCCT−3′	5′‐GGCGTCCTGGACCCATTT−3′	1.929	72
CD68	5′‐GTCCTGCTACCACCACCAGT−3′	5′‐GCTGGGAACCATTACTCCAA−3′	1.903	177
TLR4	5′‐CTGAATCTCTACAAAATCCC−3′	5′‐CTTAATTTCGCATCTGGATA−3′	1.945	149

Data were analyzed using the advanced relative quantification method provided by the LightCycler 480 instrument ver. 2.0 software. The GAPDH gene was chosen as the reference gene to normalize the expression levels of target genes. The primer sequences are listed in Table 1.

### 2.5 Protein–protein interaction (PPI) network analyses for candidate genes

The thirteen candidate genes studied here are, as previously mentioned, known gene targets/biomarkers of adipose tissue metabolism, development and inflammation. Pre‐ and postnatal nutritional insults could potentially modify expression levels of other genes/markers, which are closely co‐regulated with these target genes. This would result in corresponding changes in protein–metabolite abundance in different tissues. In order to reveal such regulatory and interactive networks that include our target genes, protein–protein interaction (PPI) networks were built by anchoring the target genes and deriving known and predicted interactions by running the network analyses tool STRING (Search Tool for the Retrieval of Interacting Genes/Proteins; http://string.embl.de/; (Jensen et al., 2009) which accesses a database of known and predicted protein interactions and uses one protein per gene, in the same way as described previously (Kadarmideen & Janss, 2007). If there was more than one isoform per gene, the longest isoform was selected, unless there was information to suggest that another isoform is better annotated. The interactions revealed in STRING include direct (physical) and indirect (functional) associations derived from four sources: genomic context, high‐throughput experiments, co‐expression databases, and previous knowledge (text mining).

### 2.6 Statistical analyses

Statistical evaluation of data was performed in the SAS software (v.9.2; SAS Institute) and JMP (version 10.0; SAS Institute Inc.). Homogeneity of variance was evaluated by visual inspection of residuals plots, and normality of residuals was tested by means of quantile‐quantile plots. The data were analysed by the generalized linear mixed models (GLIMMIX procedure). The models included *fixed effects* of pre‐ and postnatal nutrition and their interaction. Within the postnatal HCHF group, a few animals (one from the NORM, two from the HIGH and two from the LOW prenatal groups) had poorer growth rates compared to other animals in their groups (body weight < 30 kg vs. >35 kg at 6 months of age), but despite their skinny appearance, such lambs slaughtered at 6‐months of age had extensive abdominal fat deposition (Khanal et al., 2014). Thus, we performed additional statistical analyses to see whether these “small skinny fat” phenotype animals showed any indications of being different from the rest of the animals within their group that displayed “normal” appearances and growth trajectories for the parameters studied. Ewe body weight and body condition score (BCS) were used to group ewes at the start of the experiment to ensure even distribution of these traits, when ewes were assigned to each of the three different maternal feeding levels. Lamb gender (first priority) and birth weight (second priority) were used to allocate lambs to each of the two different postnatal treatment groups to ensure as even a gender and birth weight distribution as possible in the two postnatal treatment groups arising from a given prenatal treatment. Therefore, to avoid any biases in variance parameters, lamb birth weight and gender, and ewe body weight and BCS were used as co‐variables. Differences in least square means (LS means) were compared by Tukey's multiple comparison test and presented results are expressed as LS means with standard error of mean (LS means ± *SEM*) unless otherwise stated. The level of significance was set at *p* < .05.

## 3 RESULTS

No significant effects were detected of gender or prenatal nutrition or interactions between pre‐ and postnatal nutrition exposures for any of the studied parameters unless explicitly stated in the following.

### 3.1 Birth weight, growth and fat deposition

Results for birth weight, growth characteristics, and fat deposition and distribution in the experimental animals have been reported elsewhere (Khanal et al., 2014). A summary of the main findings is presented here (Table 2), as it is considered necessary to allow interpretation of the results from the present study. In short, the prenatally undernourished LOW lambs had reduced birth weights compared to lambs born to HIGH and NORM dams, whereas birth weights for NORM and HIGH lambs did not differ significantly. The lambs fed the obesogenic HCHF diet postnatally attained significantly higher body weights at six months of age than the CONV fed lambs. The subcutaneous fat deposition was higher in NORM lambs compared to HIGH and LOW lambs (both absolute weight and weight expressed as percentage of body weight), although a significant difference appeared only between NORM and HIGH lambs. HIGH and LOW lambs had decreased deposition ratios of subcutaneous fat relative to both mesenteric and perirenal fat, but the prenatal diet did not influence deposition ratios of mesenteric relative to perirenal fat. The HCHF lambs had significantly higher depositions in absolute weights of subcutaneous (~5.5 fold), mesenteric (~5‐fold) and especially perirenal (~9 fold) fat compared to CONV lambs (Table 2). Similar fold increases were found also after correction for body weight (results not shown). Thus, lambs fed the HCHF diet had higher deposition ratios of perirenal fat relative to both subcutaneous and mesenteric fat than CONV lambs. The postnatal diet did not influence deposition ratios of subcutaneous relative to mesenteric fat.

**Table 2:** Effects of pre‐ and postnatal nutrition on organ and adipose tissue weights in six months old lambs

Parameters	Prenatal nutrition			Postnatal nutrition	
	HIGH (*N* = 10)	NORM (*N* = 6)	LOW (*N* = 10)	HCHF (*N* = 13)	CONV (*N* = 13)
Birth weight, kg	4.38 ± 0.15^a^	4.35 ± 0.18^a^	3.89 ± 0.15^b^	–	–
At 6 months of age:					
Body weight, kg	36.5 ± 1.05^b^	44.8 ± 1.36^a^	38.5 ± 1.05^ab^	42.3 ± 0.9^a^	37.0 ± 0.9^b^
Subcutaneous fat*, g	140 ± 16^b^	234 ± 23^a^	182 ± 16^ab^	315 ± 15^a^	57 ± 14^b^
Mesenteric fat, g	718 ± 111	845 ± 149	771 ± 112	1,279 ± 97^a^	257 ± 97^b^
Perirenal fat, g	876 ± 95	990 ± 139	1,124 ± 95	1793 ± 86^a^	198 ± 85^b^
Subcutaneous:mesenteric fat ratio	0.22 ± 0.02^b^	0.33 ± 0.05^a^	0.23 ± 0.02^b^	0.26 ± 0.03	0.24 ± 0.02
Subcutaneous:perirenal fat ratio	0.23 ± 0.04^b^	0.35 ± 0.05^a^	0.22 ± 0.03^b^	0.18 ± 0.02^b^	0.33 ± 0.03^a^

Note

Data are presented as least square means ± *SEM* (For details, see (Khanal et al., 2014)). Effects of prenatal nutrition, postnatal nutrition or gender were significant *p* < .05 if the data within a row and within the respective columns are marked by different superscripts. *SD*, sheep diet; LD, lamb diet; NORM (*N* = 6; 6 male, 0 female), normal diet fulfilling requirements for energy and protein; HIGH (*N* = 10; 4 male, 6 female), 150% of requirements for energy and 100% of requirements for protein; LOW (*N* = 10; 5 male, 5 female), 50% of requirements for energy and protein; HCHF (*n* = 13; 8 male, 5 female), high carbohydrate‐high fat diet; CONV (*N* = 13; 7 male, 6 female) conventional diet to achieve moderate and constant growth rates of appr. 225 g/day. The subcutaneous fat represents the fat layer above the *longissimus dorsii* from the right side of the animal.

### 3.2 Histology: Adipose morphology, adipocyte CSA (size) and adipose CNI (cellularity)

#### 3.2.1 Subcutaneous adipose tissue

HIGH‐CONV lambs had higher proportions in adipose tissue of adipocytes and lower proportions of collagen infiltration compared to LOW‐ and NORM‐CONV lambs (Table 3). Both HIGH‐ and LOW‐HCHF lambs had lower proportions in adipose tissue of adipocytes and higher proportions of collagen infiltration compared to NORM lambs (*p* = .0005 and .001 for interaction of pre‐ and postnatal nutrition for proportions of adipocyte and collagen, respectively). The HIGH‐CONV lambs had the smallest adipocytes followed by NORM‐ and LOW‐CONV lambs (Table 3). HIGH animals had a slightly higher degree of adipocyte hypertrophy upon exposure to a HCHF diet compared to LOW and NORM lambs, whereas adipocyte sizes increased to similar sizes in NORM‐ and LOW‐HCHF lambs during obesity development (*p* < .0001 for interaction of pre‐ and postnatal nutrition; Figure 2; Table 3). Both HIGH and LOW lambs had reduced intrinsic (nonobese) as well as obesity‐induced cellularity (lower CNIs) compared to NORM lambs. In lambs fed the HCHF diet, CNI was increased the most in NORM‐CONV, less in LOW‐HCHF and not at all in HIGH‐HCHF (*p* < .0001 for interaction of pre‐ and postnatal nutrition; Table 3). Male lambs had lower proportions in adipose tissue of adipocytes (*p* = .02), higher proportions of collagen infiltration (*p* = .04) and higher CNIs (*p < *.0001) compared to female lambs. Small skinny fat lambs had reduced adipocyte CSA (4,906 ± 120 vs. 6,755 ± 81 µm^2^; *p < *.0001) and a higher proportion in adipose tissue of micro‐vessels (5.7 ± 0.67 vs. 3.5 ± 0.44%; *p = *.03) compared to other HCHF‐fed lambs.

**Table 3:** Effects of pre‐ and postnatal nutrition and gender on adipocyte cross‐sectional area, tissue composition and cell number index in different adipose tissues

Item	HIGH‐HCHF (*N* = 5)	HIGH‐CONV (*N* = 5)	LOW‐HCHF (*N* = 5)	LOW‐CONV (*N* = 5)	NORM‐HCHF (*N* = 3)	NORM‐CONV (*N* = 3)	Male (*N* = 12)	Female (*N* = 11)	*p* values			
									*SD*	LD	SD*LD	Gender
Subcutaneous fat												
A‐CSA (µm^2^)	6,535 ± 78^a^	1,600 ± 101^e^	6,088 ± 94^b^	2,426 ± 90^c^	6,088 ± 139^b^	2,107 ± 111^d^	4,098 ± 51	4,184 ± 67	0.09	<0.0001	<0.0001	0.37
Adipocyte (%)	85.3 ± 2^ab^	90 ± 2.6^a^	81.8 ± 2.3^bc^	79.2 ± 2.3^c^	90 ± 3.5^a^	74.5 ± 2.9^c^	80.6 ± 1.3^b^	86.6 ± 1.7^a^	0.01	0.1	0.0005	0.02
Collagen (%)	9.8 ± 1.8^bcd^	5.7 ± 2.4^d^	13.5 ± 2.1^ab^	14.7 ± 2.1^abc^	6.2 ± 3.2^cd^	19.5 ± 2.6^a^	13.8 ± 1.2^a^	9.3 ± 1.6^b^	0.01	0.17	0.001	0.04
Micro‐vessels (%)	5.0 ± 0.6	4.4 ± 0.9	4.6 ± 0.8	6.1 ± 0.8	3.7 ± 1.2	6.0 ± 0.9	5.5 ± 0.4	4.4 ± 0.6	0.64	0.24	0.17	0.18
CNI	35.8 ± 0.8^c^	36.1 ± 0.8^c^	51.5 ± 1.2^b^	27.6 ± 0.8^d^	62.5 ± 1.8^a^	48.5 ± 1.4^b^	44.4 ± 0.6^a^	35.8 ± 0.6^b^	<0.0001	0.40	<0.0001	<0.0001
Mesenteric fat												
A‐CSA (µm^2^)	7,994 ± 112^b^	4,543 ± 138^c^	8,567 ± 146^a^	4,233 ± 111^c^	7,949 ± 207^b^	4,251 ± 137^c^	5,176 ± 69^b^	7,336 ± 90^a^	0.11	<0.0001	0.0007	<0.0001
Adipocyte (%)	92.6 ± 1.3^ab^	96.3 ± 1.7^a^	89 ± 1.5^bc^	89.7 ± 1.3b^c^	86.3 ± 2.1^c^	95.4 ± 1.6^a^	88.2 ± 0.7^b^	94.9 ± 1.1^a^	0.004	0.007	0.02	<0.0001
Collagen (%)	2.2 ± 1^ab^	0.6 ± 1.4^ab^	3.9 ± 1.2^a^	3.9 ± 1.1^a^	4.2 ± 1.7^ab^	0.008 ± 1.3^b^	4.6 ± 0.6^a^	0.3 ± 0.9^b^	0.1	0.15	0.22	0.0004
Micro‐vessels (%)	5.2 ± 0.7^bcd^	3.1 ± 0.9^d^	7.1 ± 0.8^b^	6.4 ± 0.7^bc^	9.5 ± 1.2^a^	4.6 ± 0.9^cd^	7.2 ± 0.4^a^	4.8 ± 0.6^b^	0.003	0.004	0.02	0.002
CNI	61.7 ± 1.4^b^	40.4 ± 0.8^e^	53.2 ± 1.3^c^	34.8 ± 0.7^f^	84.5 ± 2.7^a^	46.3 ± 1.2^d^	45.2 ± 0.7	49.9 ± 0.7	<0.0001	0.03	<0.0001	0.5
Perirenal fat												
A‐CSA (µm^2^)	13,786 ± 120^a^	3,816 ± 150^e^	11,980 ± 149^b^	2,991 ± 131^f^	7,354 ± 214^c^	5,338 ± 176^d^	7,152 ± 77^b^	7,936 ± 99^a^	<0.0001	<0.0001	<0.0001	<0.0001
Adipocyte (%)	96 ± 0.9^ab^	90.3 ± 1.1^c^	97.2 ± 1.0^a^	93.5 ± 1.0^b^	95.7 ± 1.6^ab^	94.5 ± 1.2^ab^	94.4 ± 0.6	94.7 ± 0.7	0.1	0.003	0.13	0.77
Collagen (%)	0.6 ± 0.7^b^	3.4 ± 0.9^a^	0.6 ± 0.8^b^	1.3 ± 0.8^ab^	1.3 ± 1.2^ab^	0.8 ± 1^b^	1.5 ± 0.4	1.2 ± 0.6	0.41	0.28	0.15	0.7
Micro‐vessels (%)	3.4 ± 0.5^cd^	6.3 ± 0.6^a^	2.2 ± 0.6^d^	5.2 ± 0.5^ab^	3 ± 0.8^cd^	4.6 ± 0.7^bc^	4.1 ± 0.30	4.1 ± 0.4	0.12	<0.0001	0.49	0.96
CNI	41.7 ± 0.9^c^	30.2 ± 0.6^e^	57.9 ± 1.3^b^	32.4 ± 0.7^d^	112.6 ± 3.2^a^	31.5 ± 0.9^de^	46.3 ± 0.6^a^	37.4 ± 0.5^b^	<0.0001	<0.0001	<0.0001	<0.0001
Epicardial fat												
A‐CSA (µm^2^)	6,462 ± 83^ab^	4,244 ± 111^c^	6,345 ± 93^b^	3,912 ± 81^d^	6,668 ± 145^a^	2,572 ± 141^e^	5,139 ± 51^a^	4,929 ± 66^b^	<0.0001	<0.0001	<0.0001	0.024
Adipocyte (%)	91.3 ± 1.1^b^	92.9 ± 1.6^ab^	94.7 ± 1.3^ab^	92.3 ± 1.2^ab^	93.3 ± 1.9^ab^	95.2 ± 1.5^a^	92.8 ± 0.7	93.8 ± 1	0.39	0.81	0.14	0.42
Collagen (%)	5.8 ± 1^a^	4.3 ± 1.3^ab^	2.3 ± 1.1^b^	5.5 ± 1^a^	2.4 ± 1.6^ab^	1.8 ± 1.3^b^	4.4 ± 0.6	3 ± 0.8	0.08	0.77	0.04	0.19
Micro‐vessels (%)	2.9 ± 0.5	2.8 ± 0.7	3 ± 0.6	2.2 ± 0.6	4.3 ± 0.9	3.1 ± 0.7	2.8 ± 0.3	3.2 ± 0.4	0.26	0.31	0.65	0.51

Note

Data are presented as least square means ± *SEM*. Effects of prenatal nutrition, postnatal nutrition or gender were significant *p* < .05 if the data within a row and within the respective columns (dietary treatments or gender groups) are marked by different superscripts. A‐CSA, adipocyte cross‐sectional area; CNI, cell number index calculated as adipocyte mass (total fat mass (kg) multiplied by the %’age of adipocytes in the tissue) divided by the volume of a spherical adipocyte with a radius derived from the measured mean CSA; *SD*, sheep diet; LD, lamb diet; HIGH (*N* = 10; four males, six females; diet fed to twin‐pregnant dams during the last trimester and fulfilling 150% of their daily energy and 110% of their daily protein requirements); LOW (*N* = 10; 5 males, 5 females; diet fed to twin‐pregnant dams during the last trimester and fulfilling 50% of their daily energy and protein requirements); NORM (*N* = 6; six males, 0 female; diet fed to twin‐pregnant dams during the last trimester and fulfilling 100% of their daily energy and protein requirements); HCHF (*N* = 13; 8 males, 5 females; high‐carbohydrate, high‐fat postnatal diet fed to lambs and consisting of cream‐milk replacer mix in a 1:1 ratio supplemented with rolled maize) and CONV (*N* = 13; 7 males, 6 females; conventional postnatal diet fed to lambs and consisting of milk replacer and hay until 8 weeks of age and hay only thereafter and adjusted to achieve moderate and constant growth rates of approx. 225 g/day). CNIs were not calculated for epicardial fat since it was attached to cardiac muscle and it was not possible to cut out the whole tissue.

#### 3.2.2 Mesenteric adipose tissue

LOW‐CONV lambs had a lower proportion of adipocytes and higher proportion of collagen in adipose tissue compared to HIGH‐ and NORM‐CONV lambs, and HIGH‐CONV lambs had a lower proportion of microvessels compared to LOW‐ and NORM‐CONV lambs. When fed, the obesogenic HCHF diet, HIGH lambs attained a higher proportion of adipocytes and both HIGH and LOW lambs had lower proportions of microvessels compared to NORM lambs (*p = *.02 for interaction of pre‐ and postnatal nutrition for proportions of both adipocyte and micro‐vessels). All CONV lambs, regardless of their prenatal nutrition, had similar adipocyte size (Figure 3). All HCHF fed lambs had larger sized adipocytes compared to CONV fed lambs, however LOW‐HCHF lambs attained the largest adipocyte CSA (*p = *.0007 for interaction of pre‐ and postnatal nutrition; Table 3). Both HIGH and LOW lambs had lower nonobese and obesity‐induced CNIs compared to NORM lambs. Additionally, HIGH lambs had increased intrinsic (nonobese) and obesity‐induced CNIs compared to LOW lambs (interaction of pre‐ and postnatal nutrition *p < *.0001; Table 3). Male lambs had lower adipocyte CSA (*p < *.0001), a lower proportion of adipocytes (*p < *.0001) and higher proportions of collagen (*p = *.0004) and microvessels (*p = *.002) in adipose tissue compared to female lambs. Small skinny fat lambs had higher adipocyte CSA (9,074 ± 104 vs. 8,584 ± 104 µm^2^; *p < *.001), a lower proportion of adipocytes (88.9 ± 1.41 vs. 92.7 ± 0.85%; *p* = .006), and a higher proportion of collagen (4.27 ± 0.96 vs. 1.61 ± 0.58%; *p = *.013) compared to other HCHF lambs.

#### 3.2.3 Perirenal adipose tissue

Both HIGH‐CONV and particularly LOW‐CONV lambs had lower intrinsic (nonobese) adipocyte size compared to NORM‐CONV lambs. Upon exposure to an early postnatal HCHF diet, both HIGH and LOW lambs had a much more marked increase in adipocyte size compared to NORM lambs (*p* < .0001 for interaction of pre‐ and postnatal nutrition; Figure 4; Table 3), and perirenal adipocytes in LOW‐ and HIGH‐HCHF lambs were by far the largest across all the studied tissues. The proportions of adipocytes and microvessels were increased (*p* = .003) and decreased (*p* < .001), respectively, in lambs fed the HCHF compared to CONV diet. Intrinsic cellularity (CNI) was similar in all CONV fed groups. Upon exposure to the HCHF diet, CNI of adipocytes increased in all lambs, but the obesity‐induced increase in CNI was much less pronounced in HIGH (38%) and LOW lambs (79%) compared to NORM lambs (257%), and HIGH‐HCHF had lower CNI also compared to LOW‐HCHF lambs. As in subcutaneous fat, male lambs had higher CNIs compared to female lambs (*p < *.0001). Small skinny fat lambs had lower adipocyte CSA (11,454 ± 108 vs. 12,100 ± 108 µm^2^) compared to the rest of the HCHF lambs (*p < *.0001).

#### 3.2.4 Epicardial adipose tissue

HIGH lambs had the highest intrinsic (nonobese) adipocyte size and NORM lambs the lowest with LOW lambs in between. Upon exposure to the early postnatal HCHF diet, all lambs increased their adipocyte size, but the obesity‐induced hypertrophy was less pronounced in HIGH and LOW lambs, which attained smaller adipocyte areas compared to NORM lambs (*p* < .0001 for interaction of pre‐ and postnatal nutrition; Table 3; Figure S1). Small skinny fat lambs had lower adipocyte CSA (5,593 ± 120 vs. 7,758 ± 84 µm^2^) compared to the rest of the HCHF lambs (*p < *.0001).

### 3.3 qPCR

Expression levels for selected target genes were studied in all of the three adipose tissues and covered genes involved in: (a) lipid metabolism: FASN (fatty acid synthase), FABP4 (fatty acid‐binding protein 4), LPL (lipoprotein lipase), HSL (hormone sensitive lipase), (b) glucose transport: GLUT4 (glucose transporter member 4), (c) angiogenesis: vascular endothelial growth factor A (VEGFA), (d) adipose tissue differentiation: PPAR‐γ (peroxisome proliferator‐activated receptor gamma), C/EBP‐β (CCAAT enhancer‐binding protein beta), and (e) inflammation: TNF‐α (tumor necrosis factor alpha), MCP‐1 (monocyte chemoattractant protein 1), TLR4 (toll‐like receptor 4), CD68 (cluster of differentiation 68), and IL‐6 (interleukin 6) (Ailhaud, Grimaldi, & Négrel, 1992) (Table 1).

#### 3.3.1 Subcutaneous adipose tissue

##### Prenatal effects

**Figure 5:**
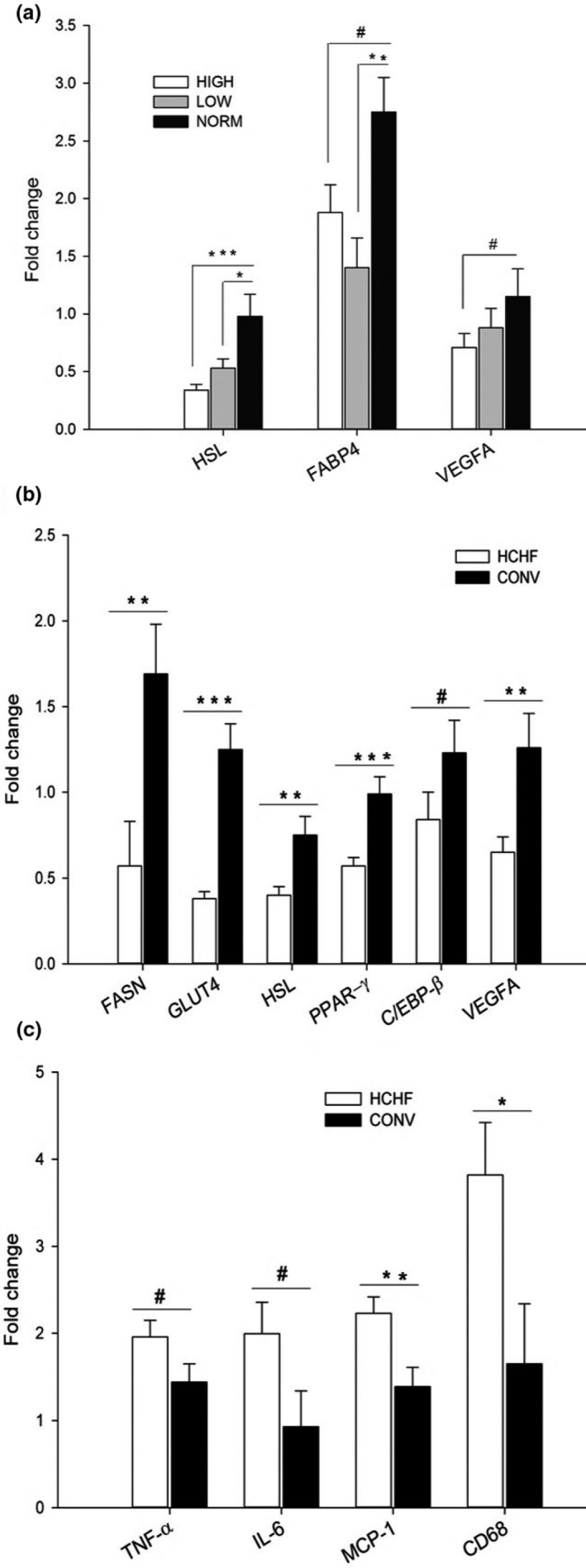
mRNA expression of genes in subcutaneous adipose tissue as affected by prenatal (a) and postnatal (b and c) nutrition. HSL, hormone sensitive lipase; FABP4, fatty acid‐binding protein 4; VEGFA, vascular endothelial growth factor A; FASN, fatty acid synthase; GLUT4, glucose transporter 4; PPAR‐γ, peroxisome proliferator‐activated receptor‐γ; C/EBP‐β, CCAAT/enhancer‐binding protein‐β; TNF‐α, tumor necrosis factor‐α; IL‐6, interleukin‐6; MCP‐1, monocyte chemoattractant protein‐1; CD68, cluster of differentiation 68. HIGH, LOW, NORM, HCHF, and CONV: See legends to Figure 1. Data represents expression ratios relative to glyceraldehyde 3‐phosphate dehydrogenase, and values are presented as least square (LS) means ± *SEM*. Bars indicate tendencies (^#^ *p < *.1) and significant differences between treatment groups (*p < .05; **p < .01; ***p < .001)

In lambs exposed to prenatal over‐ and undernutrition, mRNA expression was reduced (and to a similar extent) for genes associated with lipid metabolism compared to NORM lambs, namely HSL (*p = *.0008 and *p = *.049 for HIGH and LOW, respectively) and FABP4 (*p = *.0076 for LOW and a nonsignificant tendency for HIGH (*p = *.086); Figure 5a). A similar nonsignificant trend was observed for the angiogenic factor, VEGFA (*p = *.097).

##### Postnatal effects

The postnatal HCHF diet decreased the expression of most of the studied genes with relation to lipid metabolism and adipose development (Figure 5b). Thus, HCHF lambs had decreased mRNA expressions of FASN (~70% reduction; *p* < .01), GLUT4 (~70% reduction; *p* < .0001), HSL (*p* < .01), PPAR‐γ (*p* < .001), C/EBP‐β (*p* = .076), and VEGFA (*p* < .01) compared to CONV lambs. However, for inflammatory genes, the opposite pattern was observed, since the postnatal HCHF diet increased mRNA expressions of MCP‐1 (*p* < .01) and CD68 (>2‐fold, *p* < .05) with similar nonsignificant trends observed for TNF‐α (*p* = .072), IL‐6 (*p* = .0658) compared to CONV lambs (Figure 5c).

##### Gender effects

Female lambs had higher mRNA expression of LPL (0.77 ± 0.16 vs. 0.41 ± 0.06; *p* < .01) and TNF‐α (2.4 ± 0.23 vs. 1.30 ± 0.18; *p* = .001) genes compared to male lambs.

#### 3.3.2 Mesenteric adipose tissue

##### Prenatal effects

None of the studied genes were affected by the prenatal nutrition, except that LOW lambs exposed to the HCHF diet (1.95 ± 0.27) had higher mRNA expression of the TLR4 gene compared to LOW lambs fed the CONV diet (0.79 ± 0.27; *p < *.01), resulting in a significant interaction of pre‐ and postnatal diet for this gene (*p < *.05; Figure S2).

##### Postnatal effects

As for subcutaneous adipose tissue, the postnatal HCHF diet decreased the mRNA expression of most of the studied genes associated with lipid metabolism and adipose tissue development (Figure S2A). The HCHF lambs had reduced mRNA expression of FASN (~50% reduction; *p < *.01), FABP4 (*p* < .0001)*,* GLUT4 (~70% reduction; *p* < .0001), HSL (~75% reduction; *p* < .0001), LPL (*p* < .0001*)*, PPAR‐γ (*p < *.0001), and VEGFA (~70% reduction; *p* < .0001) with a nonsignificant trend observed also for C/EBP‐β (*p* = .092) as compared to CONV fed lambs. As for subcutaneous fat, the postnatal HCHF diet also increased mRNA expressions of the inflammatory markers TNF‐α (*p* < .001), IL‐6 (~4.5 fold, *p < *.001), MCP‐1 (~3.5 fold, *p* < .0001), and CD68 (~3.5 fold, *p* < .001) (Figure S2).

#### 3.3.3 Perirenal adipose tissue

##### Prenatal effects

**Figure 6:**
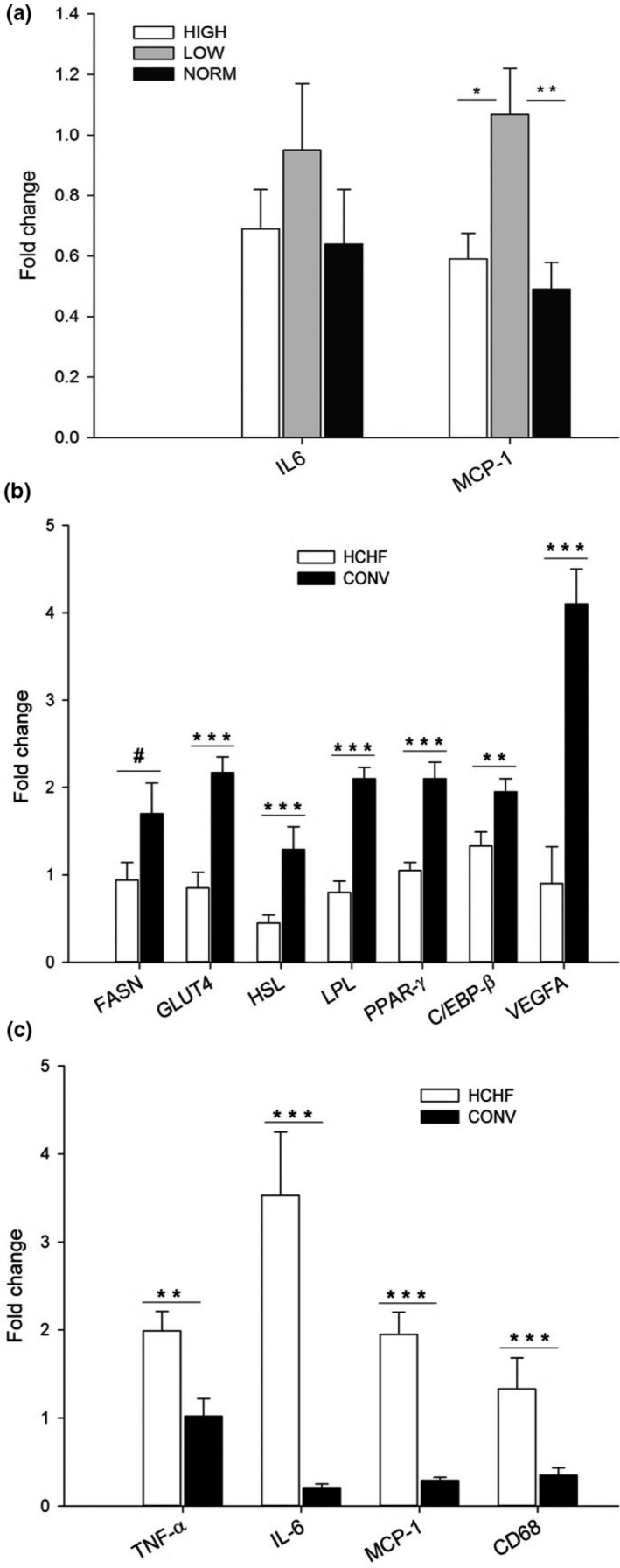
mRNA expression of genes in perirenal adipose tissue as affected by prenatal (a) and postnatal (b and c) nutrition. LPL, lipoprotein lipase. IL‐6, MCP‐1, FASN, GLUT4, HSL, PPAR‐γ, C/EBP‐β, VEGFA, TNF‐α, IL‐6, MCP‐1, C68, HIGH, NORM, LOW, HCHF and CONV: see legend to Figure 5. Data represent expression ratios relative to glyceraldehyde 3‐phosphate dehydrogenase, and values are presented as least square (LS) means ± *SEM*. Bars indicate tendencies (^#^ *p < *.1) and significant differences between treatment groups (^*^ *p* < .05; **p < .01; ***p < .001)

LOW lambs had significantly higher mRNA expression of MCP‐1 compared to HIGH (*p < *.05*)* and NORM lambs (*p < *.01), which had similar expression levels of MCP‐1 (Figure 6a). There was also a similar tendency (*p = *.058) for upregulation of mRNA expression of the IL‐6 gene in LOW compared to HIGH and NORM lambs.

##### Postnatal effects

The postnatal HCHF diet decreased the mRNA expressions for most of the genes studied related to lipid metabolism and adipose development. As in subcutaneous and mesenteric fat, the HCHF diet decreased mRNA expression in perirenal fat of FASN (*p = *.052), GLUT4 (~50% reduction; *p* < .0001), HSL (~50% reduction; *p* < .001), LPL (~50% reduction; *p < *.0001*)*, PPAR‐γ (*p* < .0001), C/EBP‐β (*p < *.01) and VEGFA (~75% reduction; *p* < .0001) (Figure 6b), but increased expressions of the inflammatory markers TNF‐α (*p* < .01), IL‐6 (~11.5 fold, *p* < .0001), MCP‐1 (~4.5 fold, *p* < .0001), and CD68 (~3‐fold, *p* < .001) (Figure 6c).

##### Gender effects

No significant gender differences were observed in the perirenal adipose tissue for any of the genes studied.

#### 3.3.4 Epicardial adipose tissue

##### Prenatal effects

**Figure 7:**
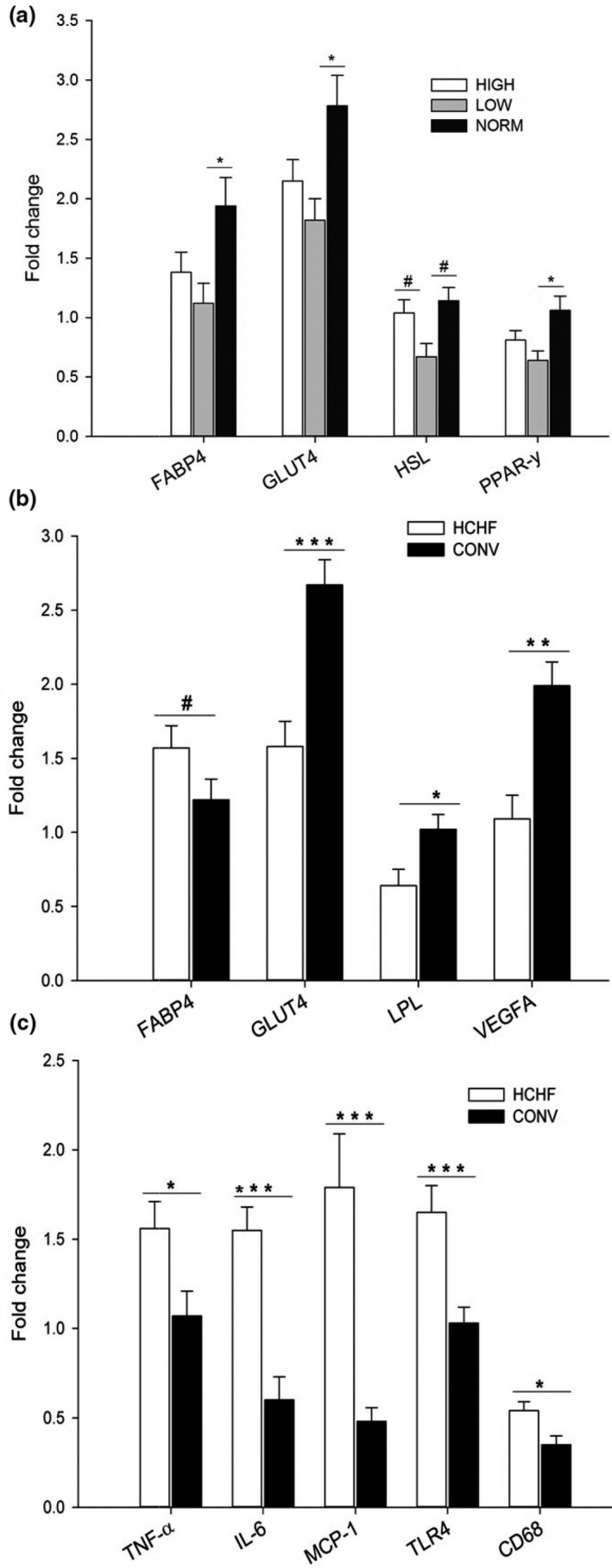
mRNA expression of genes in epicardial adipose tissue as affected by prenatal (a) and postnatal (b and c) nutrition. TLR4, toll like receptor 4. HSL, PPAR‐γ, FABP4, GLUT4, LPL, VEGFA, TNF‐α, IL‐6, MCP‐1, CD68, HIGH, NORM, LOW, HCHF, and CONV: see legends to Figures 5 and 6. Data represent expression ratios relative to glyceraldehyde 3‐phosphate dehydrogenase, and values are presented as least square (LS) means ± *SEM*. Bars indicate tendencies (^#^ *p* < .1) and significant differences between treatment groups (*p < .05; **p < .01; ***p < .001)

Epicardial adipose tissue was the most sensitive to prenatal diet effects followed by subcutaneous adipose tissue. The prenatal diet affected the mRNA expression of a range of genes involved in lipid metabolism and adipose tissue development, namely FABP4 (*p < *.05), GLUT4 (*p < *.05), HSL (*p < *.05) and PPAR‐γ (*p < *.05) (Figure 7a). Lambs exposed to LOW levels of nutrition prenatally had reduced mRNA expressions of FABP4, GLUT4, and PPAR‐γ with a tendency observed also for HSL (*p* < .05, *p < *.05, *p < *.05 and *p = *.064, respectively). Similar expression levels were observed for all the studied genes in HIGH and LOW lambs (except for the HSL gene), but the numerical reductions in expression levels for these genes in HIGH compared to CONV lambs were not significantly.

##### Postnatal effects

Similar to the subcutaneous, mesenteric and perirenal adipose tissue depots, the postnatal HCHF diet decreased the mRNA expressions in epicardial adipose tissue for most of the genes studied with relation to lipid metabolism and adipose tissue development (Figure 7b). The expression levels of GLUT4 (*p* < .001), LPL (*p < *.05) and VEGFA (~2‐fold, *p* = .001) were decreased in HCHF compared to CONV lambs (Figure 7a). As with the other adipose tissue depots studied, the opposite trend was observed for inflammatory marker genes, where the HCHF diet increased the expression of TNF‐α (*p* < .05), IL‐6 (~3‐fold, *p* < .001), MCP‐1 (~3.5 fold, *p* < .0001), TLR4 (*p < *.001), and CD68 (*p* < .05) levels as compared to CONV‐fed lambs (Figure 7c).

##### Gender effects

Female lambs had higher mRNA expressions of FASN (1.17 ± 0.16 vs. 0.60 ± 0.14; *p < *.05) and LPL (1.08 ± 0.11 vs. 0.65 ± 0.10; *p < *.05) genes compared to male lambs. On the other hand, male lambs had higher mRNA expressions of MCP‐1 (1.13 ± 0.17 vs. 0.68 ± 0.12; *p < *.05) and IL‐6 (1.42 ± 0.12 vs. 0.59 ± 0.13; *p < *.01) genes as compared to female lambs.

### 3.4 Protein‐protein interaction networks analyses

The results of the PPI networks are given in two formats: a network with varying levels of confidence (Figure S3) and a network showing evidence for interactions (Figure S4). The input nodes are colored and nodes of a higher iteration/depth are in white. Each family of proteins is assigned a different color. The STRING analyses revealed many (more than 20) other candidate genes/molecular markers that very strongly interact with the target genes/markers studied, and they included: lipid metabolism markers such as monoacylglyceride lipase (MGLL), perilipin 1 (PLIN1) and acetyl co‐enzyme A carboxylase (ACACA); markers of adipose tissue development such as sterol regulatory‐binding protein‐1 (SREBP1); and a family of apolipoproteins, such as apolipoprotein E (APOE), apolipoprotein A1 (APOA1) and apolipoprotein C3 (APOC3). Other markers such as colony‐stimulating factor 2 (CSF2, involved in cell proliferation and differentiation) and nitric oxide synthase 2 (NOS2) interacted with the inflammatory markers, which in our study were affected by the postnatal HCHF diet.

## 4 DISCUSSION

The present study confirmed our hypotheses that prenatal malnutrition, both in the form of late gestation under‐ and overnutrition, can predispose for visceral obesity in young offspring, by interfering with adipose tissue developmental and functional traits, but in a tissue‐specific manner. It confirms findings from previous studies in different animal species including sheep (Long et al., 2015), cattle (Long et al., 2012) and rats (Bayol, Simbi, Bertrand, & Stickland, 2008; Bayol, Simbi, & Stickland, 2005) that maternal malnutrition imposed at different stages of fetal development can alter the fat distribution patterns in offspring. The major new finding in this study was that (a) both prenatal over‐ and undernutrition depressed intrinsic (nonobese) adipose cellularity in subcutaneous and mesenteric adipose tissue, and reduced the ability of subcutaneous, mesenteric, and perirenal adipose tissue to expand by hyperplasic rather than hypertrophic growth, (b) this was associated with a dramatic obesity‐induced increase in perirenal adipocyte cell size, (c) long‐term consequences of prenatal malnutrition on gene expression patterns were observed exclusively in subcutaneous and epicardial fat and could not consistently be related to observed changes in morphology and expandability traits across the different adipose tissues, (d) epicardial adipose tissue, of which very little is known (Yamaguchi et al., 2015), was target of both pre‐ and postnatal nutrition, but in a distinctly different way compared to the other three adipose tissues.

### 4.1 Long‐term implications of fetal programming are differentially expressed in adipose tissues affecting both intrinsic cellularity and hyperplasic ability upon early obesity development

The long‐term consequences of fetal under‐ and overnutrition were manifested differentially on morphological changes and genes expression in the different adipose depots studied. Such differential impacts of late fetal malnutrition on adipose tissue functional development may be related to the timing of adipose tissue development (Roseboom, Rooij, & Painter, 2006) relative to the timing of the malnutritional exposure. Distinct differences in responses to late gestation malnutrition may, however, also be related to depot‐specific differences in the ability of adipogenic precursors to develop in response to different nutritional exposures (Joe, Yi, Even, Vogl, & Rossi, 2009).

In the sheep fetus, the formation of subcutaneous fat begins around day 90 of gestation followed by a significant regression from around gestation day 115, and it virtually disappears at term (~147 days in sheep) (Alexander, 1978). The formation of perirenal‐intra‐abdominal fat commences as a brown fat at around day 70 of gestation in sheep, and a rapid increase in fat mass along with a parallel increase in lipid content occurs until day 110–120 of gestation (Alexander, 1978). In fact, from ~90 to 125 days of gestation, there is significant increase (>7‐fold) in the amount of perirenal adipose tissue formed, thereafter it declines by 145 days of gestation (Symonds, Phillips, Anthony, Owens, & McMillen, 1998), and in neonates the brown tissue is rapidly replaced by white adipose tissue in the perirenal fat depot (Symonds, Bryant, Clarke, Darby, & Lomax, 1992). Thus, maternal nutrient restriction resulted in a reduced expression of growth factors and adipogenesis markers was observed in sheep in perirenal fetal adipose tissue at 89 d of gestation giving rise to lower fetal and adipose tissue fetal mass in late gestation (Wallace et al., 2015) and nutrient restriction during late gestation leads to a reduced perirenal adipose tissue weight in the sheep fetus near term (Budge et al., 2004). These studies suggest that a considerable part of subcutaneous and perirenal adipose tissue remodeling takes place during late gestation, and insults at this stage could potentially interfere with their future functional maturation and expandability.

Much less information is available on the ontogenesis of mesenteric and particularly epicardial adipose tissues. Contrary to subcutaneous adipose tissue, which develops during fetal life, visceral adipose tissue, appears to preferentially differentiate in postnatal life both in sheep and humans (Symonds, Mostyn, Pearce, Budge, & Stephenson, 2003). Knowledge regarding the ontogeny of epicardial fat formation and its function is extremely limited. Until very recently, it was believed not to exist in rodents models (Yamaguchi et al., 2015), and knowledge about this tissue is mainly derived from information provided by noninvasive imaging techniques (Wong et al., 2017). Epicardial adipose tissue has a common embryonic origin with the heart (Antonopoulos & Antoniades, 2017), and has been quantified in human fetuses by echocardiography from week 20 in pregnancy (Jackson et al., 2016). Thus, development commences early in fetal life, and adverse fetal programming predisposing for coronary heart diseases later in life in humans has in fact been linked to maternal undernutrition particularly in early gestation (Roseboom et al., 2000, 2006).

In this study, long‐term consequences of late gestation malnutrition were observed in all adipose tissues, but in a tissue‐specific way, and they will therefore be discussed individually.

#### 4.1.1 Subcutaneous adipose tissue

Fat tissue has a limited capacity to expand in response to a constant high nutrient load (Virtue & Vidal‐Puig, 2008), and the ability of adipocytes to expand can be restricted by several factors, including hypoxia and matrix mechanics (Halberg et al., 2009). Studies in humans have shown that a predisposition for central obesity in South Asians compared to Caucasians has been associated with a reduced capacity in superficial subcutaneous adipose tissue to deposit fatty acids (Sniderman et al., 2007). This led to the proposition of the “adipose tissue expandability” hypothesis, stating that limitations to the growth and expansion of subcutaneous adipose tissue will diminish its lipid storing capacity resulting in redirection of fat deposition toward visceral and ectopic regions (Sniderman et al., 2007). This can be associated with undesirable metabolic disturbances, since subcutaneous fat is considered a “healthy fat” in contrast to other adipose tissues. In fact, transplantation of subcutaneous fat into the visceral cavity of recipient mice (but not visceral fat into the subcutaneous compartment) could improve whole body insulin sensitivity and stimulate peripheral glucose uptake (Tran et al., 2008).

In this study, prenatal exposure to either over‐ or undernutrition not only decreased intrinsic cellularity of subcutaneous adipose tissue, but also restricted its hyperplasic expandability, particularly in HIGH, upon early postnatal obesity development, which made this tissue rely on hypertrophic expansion during obesity development. However, since the upper limit for hypertrophic expandability of subcutaneous adipocytes appeared to be fixed (in contrast to the other tissues studied), a reduction in hyperplasic ability due to malnutrition in late fetal life, in effect caused a reduction in the overall expandability of this tissue and increased proportions of fat deposited in the abdominal region (Table 1).

Increased or decreased intake of protein during pregnancy have also been found to reduce subcutaneous fat mass in piglets, which in agreement with our study could be associated with reduced subcutaneous adipocyte numbers, but not size (Rehfeldt et al., 2012). Thus, prenatal nutrition appears to interfere with subcutaneous adipose tissue cellularity and hence expandability, and this will alter regional preferences for fat distribution patterns later in life (reviewed by (Lecoutre & Breton, 2014)).

We examined changes in gene expression levels for markers known to be involved in adipocyte differentiation, regulation of lipid and carbohydrate metabolism, angiogenesis and inflammation. Lower expression of lipid metabolism (FAB4 and HSL) and angiogenic (VEGF) genes were observed in both fetally under‐ and overnourished lambs and this could be part of the underlying reason for reduced intrinsic subcutaneous adipocyte cellularity. The development and expansion of adipocytes require a supply of oxygen and nutrients from blood vessels, and this is achieved by development of the capillary network through angiogenesis (Moreno‐Indias & Tinahones, 2015). Reduced adipocyte differentiation and fat mobilization in addition to poor angiogenesis, can lower the ability of the subcutaneous adipose tissue to store lipids (Ortega et al., 2010; Sun et al., 2012).

Thus, not only undernutrition but also overnutrition, during the last trimester can reduce subcutaneous expandability as a result of reduced intrinsic cellularity and reduced ability for hyperplasic growth during obesity development. These must be important determinants for expandability, when the upper limit for adipocyte cell size apparently is rather fixed in subcutaneous tissue, as suggested by our results. Changes in gene expressions suggest that reduced capacity for angiogenesis and lipid metabolism may be part of the underlying mechanism.

#### 4.1.2 Perirenal adipose tissue

Perirenal adipose tissue expandability traits are also affected by abnormal maternal nutrition during pregnancy, but in a different way than for subcutaneous adipose tissue. Thus, in rat offspring from dams fed a low‐calorie diet during gestation, perirenal adipocyte sizes were increased along with intra‐abdominal fat accumulation, when they were challenged to a hypercaloric diet in postnatal life (Bieswal et al., 2006). In this study, we observed extreme hypertrophy in both LOW and HIGH lambs exposed to the obesogenic HCHF diet in early postnatal life as compared to NORM‐HCHF lambs, and this was associated with impaired ability to upregulate cell numbers during obesity development. Adipocyte size is positively correlated to secretion of adipokines and inflammatory cytokines, such as leptin, interleukin‐6 and −8, and tumor necrosis factor alpha (TNF‐α) (Skurk, Alberti‐Huber, Herder, & Hauner, 2007; Suganami, Nishida, & Ogawa, 2005). This can lead to infiltration of macrophages and inflammation of adipose tissue (Lecoutre & Breton, 2014), and in a previous sheep study, prenatal undernutrition increased the expression of pro‐inflammatory gene markers in perirenal adipose tissue in offspring (Sharkey et al., 2009). It is possible that the fetal origin of such a hypertrophy‐driven pro‐inflammatory response in perirenal adipose tissue may particularly be the results of a prenatal undernutrition exposure, since only LOW (but not HIGH) lambs in our study had upregulation of IL‐6 and MCP‐1 gene expression, when exposed to the obesogenic HCHF diet postnatally. Interestingly, subgroups of animals from all treatment groups were kept after 6 months of age and transferred to a normal low‐fat sheep diet, which they were fed for 2 years, and then they were studied as adults at 2.6 years of age. The 2.6‐year‐old mismatching LOW‐HCHF sheep in contrast to all other groups showed signs of adult hypercholesterolemia, hypercreatinemia, and hyperuremia (Khanal et al., 2016). An ultrasonographic study with adult humans from the Uberlândia Heart Study (Roever et al., 2015) demonstrated that perirenal fat deposition was adversely associated with both the metabolic syndrome and cardiovascular risk factors. Taking this into consideration, it is tempting to speculate that perirenal adipose tissue may be an important target for prenatal malnutrition, and this can contribute to explain the fetal origin of the metabolic syndrome and cardiovascular disease.

Perirenal adiposity is also believed to play an important role in development of renal disorders and chronic kidney disease (Lamacchia et al., 2011; Roever et al., 2015). Due to the distinct feature of perirenal adipose tissue with a surrounding outer multi‐layered fibrous membrane and the proximity to the kidney, its expansion can directly affect kidney development and function by compression of the kidney and its blood supply and by direct exchange of adipokines and cytokines, formed in adipocytes as they enlarge (Liu, Sun, & Kong, 2018; Williams et al., 2007). Thus, the pattern of expandability in this adipose tissue (hypertrophic vs. hyperplasic) has implications for kidney function and other disorders. This is evidenced by findings in diabetic Zucker rats, where improved insulin‐sensitivity and normo‐lipidaemia were obtained by treatment with pioglitazone, and this was associated with an increase in fat mass in various adipose tissues (although perirenal adipose tissue was not studied) in an apparently healthy way by formation of new small adipocytes at the expense of shrinkage/disappearance of existing mature ones (de Souza et al., 2001).

Therefore, in individuals exposed to fetal over‐ or undernutrition, reduced subcutaneous expandability combined with impaired ability to expand perirenal fat mass by hyperplasic rather than hypertrophic growth may be a contributing risk factor for development of kidney as well as metabolic and cardiovascular disorders later in life. None of the studied genetic markers for adipose metabolic function, adipogenesis or angiogenesis could account for this prenatal impact on perirenal expandability.

#### 4.1.3 Mesenteric adipose tissue

Fetal malnutrition both in the form of under‐ and overnutrition reduced not only subcutaneous, but also mesenteric adipose intrinsic cellularity as well as the ability of both perirenal and mesenteric adipose tissue to expand by hyperplasic rather than hypertrophic growth during obesity development. Male offspring from mice fed a diet with a relatively high fat content (45% fat) during gestation were also found to have larger sized mesenteric adipocytes and enhanced expression of proinflammatory macrophage markers in mesenteric fat (Umekawa et al., 2015). In cattle, it was similarly found that adipocytes in both subcutaneous, perirenal, and mesenteric adipose tissue had larger diameters in calves subjected to undernutrition during fetal life as compared to controls (Long et al., 2012). In our study, the adverse changes in adipose expandability traits appeared to be less pronounced in fetally overnourished (HIGH) as compared to undernourished (LOW) lambs. Whether this reflects an adaptive advantage during obesity development for individuals exposed to fetal overnutrition, remains to be established. It is, however, interesting in light of the observation that only LOW‐HCHF sheep developed signs of hypercholesterolemia in adult life, as mentioned above, and mesenteric fat thickness has been identified as an independent determinant of metabolic syndrome and cardiovascular disease risk in humans (Liu, Chan, Chan, Chan, & Chu, 2006). We were unable to document any impact of fetal nutrition history on expression levels for any of the genetic markers in mesenteric adipose tissue, and the underlying reason for the fetally derived morphological changes in this tissue therefore remains to be established.

Reduced capacity of subcutaneous adipose tissue to expand, and impaired ability of both subcutaneous and perirenal adipose tissue to recruit or form new adipocytes have been identified as factors that alter preferences for fat distribution toward the abdominal region (Drolet et al., 2008). Due to its inaccessibility, it was only recently that mesenteric adipocyte size distribution was characterized for the first time in humans. The study showed that insulin resistance and type 2 diabetes in obese humans could be linked to a limited ability to form new adipocytes in this tissue (Fang, Guo, Zhou, Stahl, & Grams, 2015). Our sheep study demonstrates that malnutrition in late fetal life interferes with intrinsic cellularity and impairs the ability of all these three adipose tissues to expand by healthy hyperplasia in times of nutrient excess. This increases the risk of nutrient overflow toward the abdominal region, since upper‐hypertrophic limits for adipocyte expansion apparently are reached earlier in subcutaneous (least expandable) than mesenteric and particularly perirenal (most expandable) adipocytes.

#### 4.1.4 Epicardial adipose tissue

As previously pointed out, our knowledge about the functional development of epicardial adipose tissue is limited due to the inaccessibility of this tissue in humans and its virtual absence in rodent animal models. Hitherto, it has therefore been unknown, if adverse exposures in prenatal life can affect the functional development of epicardial tissue (Symonds, Bloor, Ojha, & Budge, 2017). To our surprise, we found that epicardial adipose tissue was the most sensitive toward prenatal malnutrition in terms of gene expression for markers of adipocyte development and lipid metabolism. Epicardial adipose tissue is associated with the development of a wide range of cardiovascular diseases in humans (Alexopoulos et al., 2010; Wong et al., 2017), and unfavorable cardiometabolic risk factors in children (Schusterova, Leenen, Jurko, Sabol, & Takacova, 2014). Epicardial fat has a high capacity for fatty acid synthesis, uptake and release, and is believed to play a special lipo‐protective role against elevated levels of free fatty acids in the coronary arterial circulation, and reversely it can act as a local energy supply for cardiac muscle through release of fatty acids when needed (Marchington, Mattacks, & Pond, 1989; Marchington & Pond, 1990). We found that prenatally malnourished lambs, both under‐ and overnourished, had higher intrinsic cross‐sectional areas of epicardial adipocytes, but their upper‐hypertrophic expandability during obesity development was reduced. This is in agreement with reduced expression of the FABP4, GLUT4, PPAR‐γ, and HSL genes. In this experiment, we did not attempt to excise the total epicardial fat mass, and to what extent these gene expression changes reflect ability of epicardial adipose tissue to store fat and hence protect the myocardium against toxic levels of circulating free fatty acids, remains to be established. Recently, it has been reviewed that complex bidirectional pathways exist, whereby epicardial‐derived adipokines and cytokines can affect cardiac function, and vice‐versa via paracrine signaling from the heart (Antonopoulos & Antoniades, 2017). We have shown for the first time that epicardial adipose tissue is another important adipose target of prenatal nutritional programming, and this can be part of the underlying reason for fetal origins of cardiovascular health disorders later in life.

### 4.2 A postnatal obesogenic diet targets all visceral depots, downregulates the expression of genes related to lipid metabolism, and is a risk factor for adipose tissue inflammation

The early postnatal obesogenic HCHF diet‐induced adipocyte hypertrophy in all studied tissues and increased adipose cellularity (not evaluated in epicardial). In agreement with this, the fat mass in HCHF compared to CONV lambs increased ~5‐fold in mesenteric and ~9‐fold in perirenal fat as previously reported (Khanal et al., 2014). This study accentuates that postnatal obesity development rely on both hypertrophic and hyperplasic adipose expansion. In our lambs, subcutaneous and epicardial adipocytes appeared to have the lowest upper‐hypertrophic limits for adipocyte expansion followed by mesenteric adipocytes, and the prenatal nutrition history did not have an impact on this upper limit despite the fetal impact hyperplasic expandability (CNI) during obesity development. In contrast, the upper limit for perirenal adipocyte expandability was enormous in the two prenatally malnourished HCHF groups (LOW‐HCHF and HIGH‐HCHF), where ability to expand by hyperplasic growth was impaired. This extreme hypertrophic expandability may have implications for kidney function in relation to obesity development as perirenal adipose tissue expansion can induce renal damage due to increasing intrarenal pressure and release of cytokines from hypertrophic adipocytes. Interestingly, we observed that the lambs fed the HCHF diet had approximately 1/3 lower kidney weights than CONV fed lambs (Khanal et al., 2014), and in this context it been shown that para‐ and perirenal fat thickness were much more closely related to renal function indices compared to other measures (Lamacchia et al., 2011).

In the progression of obesity, rat studies have suggested that hypertrophic growth of adipose tissue occurs prior to hyperplasia (Faust, Johnson, Stern, & Hirsch, 1978). Likewise, a study in obese humans suggested that once an upper‐hypertrophic limit for adipocyte expansion has been reached, further adipose expansion rely on formation of new cells (Fang et al., 2015). The upper‐hypertrophic limit is tissue dependent, and was slightly higher in subcutaneous than mesenteric fat in the human study, whereas we found the opposite in sheep. Increments in adipocyte size already reached a plateau in mice epididymal fat after 3‐weeks of high‐fat feeding, and that was associated with an upregulation of expression of lipid metabolism associated genes (Li, Yu, Pan, & Unger, 2002). This was in contrast to our study, where gene expression for markers known to be involved in glucose and fatty acid uptake, lipid metabolism, adipogenesis, and angiogenesis (Kliewer et al., 1997; Tanaka, Yoshida, Kishimoto, & Akira, 1997; Zuo, Qiang, & Farmer, 2006) were downregulated in the lambs fed the obesogenic HCHF diet. Animal species, differences between adipose tissues and dietary exposure time may explain the contrasting results. In studies with moderately obese women it has been shown, in agreement with our sheep study, that gene expression for markers of lipogenesis was progressively downregulated in subcutaneous adipose tissues as body mass index increased, whereas inflammatory marker expressions were increased in both subcutaneous and mesenteric adipose tissues (Guiu‐Jurado et al., 2015). Hence, a positive correlation has been established between adipose tissue inflammation and adipocyte size in humans (Skurk et al., 2007).

A previous mice study showed an increased expression in proinflammatory genes as obesity progressed, especially genes such as MCP‐1, MIP‐1α, and CD68, which indicate macrophage infiltration (Xu et al., 2003) and progression of obesity (Weisberg et al., 2003; Xu et al., 2003), leading to chronic low‐grade adipose tissue inflammation (Chawla, Nguyen, & Goh, 2011). Thus, an early postnatal high‐fat diet causing extensive adipocyte hypertrophy is accompanied by an increased proinflammatory response, thus giving rise to metabolic complications associated with increasing visceral fat deposition (Liu, Mei, Yang, & Li, 2014; Wronkowitz, Romacho, Sell, & Eckel, 2014).

## 5 CONCLUSIONS

In conclusion, both prenatal over‐ and undernutrition predisposed for development of abdominal adiposity and extreme perirenal hypertrophy by depressing nonobese cellularity in subcutaneous and mesenteric fat and by impairing subcutaneous, mesenteric, and perirenal adipose tissues hyperplasic (healthy) as opposed to hypertrophic (unhealthy) expandability upon exposure to a high‐fat, obesity inducing diet postpartum. The prenatal programming did not target the expression of proinflammatory markers, except that late gestation undernutrition, but not overnutrition, enhanced inflammatory responses in the perirenal adipose tissue. However, how this relates to perirenal and visceral adiposity risks remains to be established. The early postnatal HCHF diet led to hypertrophic expansion of fat mass, particularly in perirenal adipose tissue, and led to upregulated inflammatory responses in all adipose tissue depots studied. Future studies are needed to reveal, whether there are long‐term impacts of such pre‐ and postnatal dietary impacts on adipose tissue cellularity.

## AUTHOR CONTRIBUTIONS

Conceptualization: PK and MON. Data curation: PK and MON. Formal analysis: PK, DP, SS, HNK, and MON. Funding acquisition: MON. Investigation: PK and MON. Methodology: PK, DP, SBA, and MON. Project administration: MON. Ressources: MON. Supervision: MON. Writing – original draft: PK. Writing – review and editing: PK, DP, SBA, SS, HNK, and MON.

## References

[phy214359-bib-0001] Abràmoff, M. D. , Magalhães, P. J. , & Ram, S. J. (2004). Image processing with ImageJ. Biophotonics Internship, 11, 36–42.

[phy214359-bib-0002] Ailhaud, G. , Grimaldi, P. , & Négrel, R. (1992). Cellular and molecular aspects of adipose tissue development. Annual Review of Nutrition, 12, 207–233. 10.1146/annurev.nu.12.070192.001231 1503804

[phy214359-bib-0003] Alexander, G. (1978). Quantitative development of adipose tissue in foetal sheep. Australian Journal of Biological Sciences, 31, 489–503. 10.1071/BI9780489 751628

[phy214359-bib-0004] Alexopoulos, N. , McLean, D. S. , Janik, M. , Arepalli, C. D. , Stillman, A. E. , & Raggi, P. (2010). Epicardial adipose tissue and coronary artery plaque characteristics. Atherosclerosis, 210, 150–154. 10.1016/j.atherosclerosis.2009.11.020 20031133

[phy214359-bib-0005] Antonopoulos, A. S. , & Antoniades, C. (2017). The role of epicardial adipose tissue in cardiac biology: Classic concepts and emerging roles. Journal of Physiology, 595, 3907–3917. 10.1113/JP273049 28191635 PMC5471417

[phy214359-bib-0006] Bayol, S. , Simbi, B. , Bertrand, J. , & Stickland, N. (2008). Offspring from mothers fed a 'junk food' diet in pregnancy and lactation exhibit exacerbated adiposity that is more pronounced in females. Journal of Physiology, 586, 3219–3230. 10.1113/jphysiol.2008.153817 18467362 PMC2538787

[phy214359-bib-0007] Bayol, S. A. , Simbi, B. H. , & Stickland, N. C. (2005). A maternal cafeteria diet during gestation and lactation promotes adiposity and impairs skeletal muscle development and metabolism in rat offspring at weaning. Journal of Physiology, 567, 951–961. 10.1113/jphysiol.2005.088989 16020464 PMC1474235

[phy214359-bib-0008] Bieswal, F. , Ahn, M.‐T. , Reusens, B. , Holvoet, P. , Raes, M. , Rees, W. D. , & Remacle, C. (2006). The importance of catch‐up growth after early malnutrition for the programming of obesity in male rat. Obesity, 14, 1330–1343. 10.1038/oby.2006.151 16988075

[phy214359-bib-0009] Budge, H. , Edwards, L. J. , McMillen, I. C. , Bryce, A. , Warnes, K. , Pearce, S. , … Symonds, M. E. (2004). Nutritional manipulation of fetal adipose tissue deposition and uncoupling protein 1 messenger RNA abundance in the sheep: Differential effects of timing and duration. Biology of Reproduction, 71, 359–365. 10.1095/biolreprod.103.018986 15056567

[phy214359-bib-0010] Chawla, A. , Nguyen, K. D. , & Goh, Y. P. (2011). Macrophage‐mediated inflammation in metabolic disease. Nature Reviews Immunology, 11, 738–749. 10.1038/nri3071 PMC3383854 21984069

[phy214359-bib-0011] Cleal, J. K. , Poore, K. R. , Boullin, J. P. , Khan, O. , Chau, R. , Hambidge, O. , … Green, L. R. (2007). Mismatched pre‐ and postnatal nutrition leads to cardiovascular dysfunction and altered renal function in adulthood. Proceedings of the National Academy of Sciences, 104, 9529–9533. 10.1073/pnas.0610373104 PMC1890528 17483483

[phy214359-bib-0012] de Souza, C. J. , Eckhardt, M. , Gagen, K. , Dong, M. , Chen, W. , Laurent, D. , & Burkey, B. F. (2001). Effects of pioglitazone on adipose tissue remodeling within the setting of obesity and insulin resistance. Diabetes, 50, 1863–1871. 10.2337/diabetes.50.8.1863 11473050

[phy214359-bib-0013] Desai, M. , & Ross, M. G. (2011). Fetal programming of adipose tissue: Effects of intrauterine growth restriction and maternal obesity/high‐fat diet. Seminars in Reproductive Medicine, 29, 237–245. 10.1055/s-0031-1275517 21710399 PMC4010300

[phy214359-bib-0014] Drolet, R. , Richard, C. , Sniderman, A. , Mailloux, J. , Fortier, M. , Huot, C. , … Tchernof, A. (2008). Hypertrophy and hyperplasia of abdominal adipose tissues in women. International Journal of Obesity, 32, 283 10.1038/sj.ijo.0803708 17726433

[phy214359-bib-0015] Fang, L. , Guo, F. , Zhou, L. , Stahl, R. , & Grams, J. (2015). The cell size and distribution of adipocytes from subcutaneous and visceral fat is associated with type 2 diabetes mellitus in humans. Adipocyte., 4, 273–279. 10.1080/21623945.2015.1034920 26451283 PMC4573191

[phy214359-bib-0016] Faust, I. M. , Johnson, P. R. , Stern, J. S. , & Hirsch, J. (1978). Diet‐induced adipocyte number increase in adult rats: A new model of obesity. American Journal of Physiology, 235, E279–286. 10.1152/ajpendo.1978.235.3.E279 696822

[phy214359-bib-0017] Ford, S. , Hess, B. , Schwope, M. , Nijland, M. , Gilbert, J. , Vonnahme, K. , … Nathanielsz, P. (2007). Maternal undernutrition during early to mid‐gestation in the ewe results in altered growth, adiposity, and glucose tolerance in male offspring 1. Journal of Animal Science, 85, 1285–1294. 10.2527/jas.2005-624 17224460

[phy214359-bib-0018] Giblin, L. , Darimont, C. , Leone, P. , McNamara, L. B. , Blancher, F. , Berry, D. , … Lawlor, P. G. (2015). Offspring subcutaneous adipose markers are sensitive to the timing of maternal gestational weight gain. Reproductive Biology and Endocrinology, 13, 16 10.1186/s12958-015-0009-0 25879645 PMC4363193

[phy214359-bib-0019] Gluckman, P. D. , Hanson, M. A. , & Spencer, H. G. (2005). Predictive adaptive responses and human evolution. Trends in Ecology & Evolution, 20, 527–533. 10.1016/j.tree.2005.08.001 16701430

[phy214359-bib-0020] Guan, H. , Arany, E. , van Beek, J. P. , Chamson‐Reig, A. , Thyssen, S. , Hill, D. J. , & Yang, K. (2005). Adipose tissue gene expression profiling reveals distinct molecular pathways that define visceral adiposity in offspring of maternal protein‐restricted rats. American Journal of Physiology. Endocrinology and Metabolism, 288, E663–E673. 10.1152/ajpendo.00461.2004 15562247

[phy214359-bib-0021] Guiu‐Jurado, E. , Auguet, T. , Berlanga, A. , Aragonès, G. , Aguilar, C. , Sabench, F. , … Richart, C. (2015). Downregulation of de novo Fatty acid synthesis in subcutaneous adipose tissue of moderately obese women. International Journal of Molecular Sciences, 16, 29911–29922. 10.3390/ijms161226206 26694359 PMC4691149

[phy214359-bib-0022] Gundersen, H. J. G. , Bendtsen, T. F. , Korbo, L. , Marcussen, N. , Møller, A. , Nielsen, K. , … West, M. J. (1988). Some new, simple and efficient stereological methods and their use in pathological research and diagnosis. Apmis., 96, 379–394. 10.1111/j.1699-0463.1988.tb05320.x 3288247

[phy214359-bib-0023] Halberg, N. , Khan, T. , Trujillo, M. E. , Wernstedt‐Asterholm, I. , Attie, A. D. , Sherwani, S. , … Magalang, U. J. (2009). Hypoxia‐inducible factor 1α induces fibrosis and insulin resistance in white adipose tissue. Molecular and Cellular Biology, 29, 4467–4483. 19546236 10.1128/MCB.00192-09 PMC2725728

[phy214359-bib-0024] Hou, L. , Kongsted, A. H. , Ghoreishi, S. M. , Takhtsabzy, T. K. , Friedrichsen, M. , Hellgren, L. I. , … Nielsen, M. O. (2013). Pre‐ and early‐postnatal nutrition modify gene and protein expressions of muscle energy metabolism markers and phospholipid Fatty Acid composition in a muscle type specific manner in sheep. PLoS ONE, 8, e65452 10.1371/journal.pone.0065452 23755234 PMC3675032

[phy214359-bib-0025] Jackson, D. , Deschamps, D. , Myers, D. , Fields, D. , Knudtson, E. , & Gunatilake, R. (2016). Fetal epicardial fat thickness in diabetic and non‐diabetic pregnancies: A retrospective cross‐sectional study. Obesity (Silver Spring, Md.), 24, 167–171. 10.1002/oby.21353 26638197

[phy214359-bib-0026] Jensen, L. J. , Kuhn, M. , Stark, M. , Chaffron, S. , Creevey, C. , Muller, J. , … von Mering, C. (2009). STRING 8–a global view on proteins and their functional interactions in 630 organisms. Nucleic Acids Research, 37, D412–416. 10.1093/nar/gkn760 18940858 PMC2686466

[phy214359-bib-0027] Joe, A. W. B. , Yi, L. , Even, Y. , Vogl, A. W. , & Rossi, F. M. V. (2009). Depot‐specific differences in adipogenic progenitor abundance and proliferative response to high‐fat diet. Stem Cells, 27, 2563–2570. 10.1002/stem.190 19658193

[phy214359-bib-0028] Kadarmideen, H. N. , & Janss, L. L. (2007). Population and systems genetics analyses of cortisol in pigs divergently selected for stress. Physiological Genomics, 29, 57–65. 10.1152/physiolgenomics.00144.2006 17132818

[phy214359-bib-0029] Kayser, B. D. , Goran, M. I. , & Bouret, S. G. (2015). Perinatal overnutrition exacerbates adipose tissue inflammation caused by high‐fat feeding in C57BL/6J mice. PLoS ONE, 10, e0121954 10.1371/journal.pone.0121954 25835281 PMC4383546

[phy214359-bib-0030] Khanal, P. , Husted, S. V. , Axel, A. M. , Johnsen, L. , Pedersen, K. L. , Mortensen, M. S. , … Nielsen, M. O. (2014). Late gestation over‐ and undernutrition predispose for visceral adiposity in response to a post‐natal obesogenic diet, but with differential impacts on glucose‐insulin adaptations during fasting in lambs. Acta Psychologica, 210, 110–126. 10.1111/apha.12129 23746217

[phy214359-bib-0031] Khanal, P. , Johnsen, L. , Axel, A. M. D. , Hansen, P. W. , Kongsted, A. H. , Lyckegaard, N. B. , & Nielsen, M. O. (2016). Long‐term impacts of foetal malnutrition followed by early postnatal obesity on fat distribution pattern and metabolic adaptability in adult sheep. PLoS ONE, 11, e0156700 10.1371/journal.pone.0156700 27257993 PMC4892656

[phy214359-bib-0032] Kliewer, S. A. , Sundseth, S. S. , Jones, S. A. , Brown, P. J. , Wisely, G. B. , Koble, C. S. , … Lehmann, J. M. (1997). Fatty acids and eicosanoids regulate gene expression through direct interactions with peroxisome proliferator‐activated receptors alpha and gamma. Proceedings of the National Academy of Sciences of the United States of America, 94, 4318–4323. 9113987 10.1073/pnas.94.9.4318 PMC20720

[phy214359-bib-0033] Lamacchia, O. , Nicastro, V. , Camarchio, D. , Valente, U. , Grisorio, R. , Gesualdo, L. , & Cignarelli, M. (2011). Para‐ and perirenal fat thickness is an independent predictor of chronic kidney disease, increased renal resistance index and hyperuricaemia in type‐2 diabetic patients. Nephrology, Dialysis, Transplantation, 26, 892–898. 10.1093/ndt/gfq522 20798120

[phy214359-bib-0034] Lecoutre, S. , & Breton, C. (2014). The cellularity of offspring's adipose tissue is programmed by maternal nutritional manipulations. Adipocyte., 3, 256–262. 10.4161/adip.29806 26317049 PMC4550685

[phy214359-bib-0035] Li, J. , Yu, X. , Pan, W. , & Unger, R. H. (2002). Gene expression profile of rat adipose tissue at the onset of high‐fat‐diet obesity. American Journal of Physiology. Endocrinology and Metabolism, 282, E1334–E1341. 10.1152/ajpendo.00516.2001 12006364

[phy214359-bib-0036] Liu, B. X. , Sun, W. , & Kong, X. Q. (2018). Perirenal fat: A unique fat pad and potential target for cardiovascular disease. Angiology, 70(7), 584–593. 10.1177/0003319718799967 30301366

[phy214359-bib-0037] Liu, K. H. , Chan, Y. L. , Chan, W. B. , Chan, J. C. , & Chu, C. W. (2006). Mesenteric fat thickness is an independent determinant of metabolic syndrome and identifies subjects with increased carotid intima‐media thickness. Diabetes Care, 29, 379–384. 10.2337/diacare.29.02.06.dc05-1578 16443891

[phy214359-bib-0038] Liu, L. , Mei, M. , Yang, S. , & Li, Q. (2014). Roles of chronic low‐grade inflammation in the development of ectopic fat deposition. Mediators of Inflammation, 2014, 1–7. 10.1155/2014/418185 PMC4131072 25143667

[phy214359-bib-0039] Long, N. M. , Rule, D. C. , Tuersunjiang, N. , Nathanielsz, P. W. , & Ford, S. P. (2015). Maternal obesity in sheep increases fatty acid synthesis, upregulates nutrient transporters, and increases adiposity in adult male offspring after a feeding challenge. PLoS ONE, 10, e0122152 10.1371/journal.pone.0122152 25875659 PMC4398357

[phy214359-bib-0040] Long, N. M. , Tousley, C. B. , Underwood, K. R. , Paisley, S. I. , Means, W. J. , Hess, B. W. , … Ford, S. P. (2012). Effects of early‐ to mid‐gestational undernutrition with or without protein supplementation on offspring growth, carcass characteristics, and adipocyte size in beef cattle. Journal of Animal Science, 90, 197–206. 10.2527/jas.2011-4237 21908644

[phy214359-bib-0041] Marchington, J. M. , Mattacks, C. A. , & Pond, C. M. (1989). Adipose tissue in the mammalian heart and pericardium: Structure, foetal development and biochemical properties. Comparative Biochemistry and Physiology Part B: Comparative Biochemistry, 94, 225–232. 10.1016/0305-0491(89)90337-4 2591189

[phy214359-bib-0042] Marchington, J. M. , & Pond, C. M. (1990). Site‐specific properties of pericardial and epicardial adipose tissue: The effects of insulin and high‐fat feeding on lipogenesis and the incorporation of fatty acids in vitro. International Journal of Obesity, 14, 1013–1022. 2086494

[phy214359-bib-0043] Moreno‐Indias, I. , & Tinahones, F. J. (2015). Impaired adipose tissue expandability and lipogenic capacities as ones of the main causes of metabolic disorders. Journal of Diabetes Research, 2015, 1–12. 10.1155/2015/970375 PMC4398959 25922847

[phy214359-bib-0044] Muhlhausler, B. S. , Duffield, J. , & McMillen, I. C. (2007a). Increased maternal nutrition stimulates peroxisome proliferator activated receptor‐γ, adiponectin, and leptin messenger ribonucleic acid expression in adipose tissue before birth. Endocrinology, 148, 878–885. 10.1210/en.2006-1115 17068138

[phy214359-bib-0045] Muhlhausler, B. S. , Duffield, J. A. , & McMillen, I. C. (2007b). Increased maternal nutrition increases leptin expression in perirenal and subcutaneous adipose tissue in the postnatal lamb. Endocrinology, 148, 6157–6163. 10.1210/en.2007-0770 17884936

[phy214359-bib-0046] Nielsen, M. O. , Kongsted, A. H. , Thygesen, M. P. , Strathe, A. B. , Caddy, S. , Quistorff, B. , … Johnsen, L. (2013). Late gestation undernutrition can predispose for visceral adiposity by altering fat distribution patterns and increasing the preference for a high‐fat diet in early postnatal life. British Journal of Nutrition, 109, 2098–2110. 10.1017/S0007114512004199 23069212

[phy214359-bib-0047] NRC (2007). Nutrient requirements of small ruminants: Sheep, goats, cervids, and new world camelids. Washington, D.C: National Research Council of the National Academies, The National Academies Press.

[phy214359-bib-0048] Ojha, S. , Saroha, V. , Symonds, M. E. , & Budge, H. (2013). Excess nutrient supply in early life and its later metabolic consequences. Clinical and Experimental Pharmacology and Physiology, 40, 817–823. 10.1111/1440-1681.12061 23350968

[phy214359-bib-0049] Ortega, F. J. , Moreno‐Navarrete, J. M. , Pardo, G. , Sabater, M. , Hummel, M. , Ferrer, A. , … Fernández‐Real, J. M. (2010). MiRNA expression profile of human subcutaneous adipose and during adipocyte differentiation. PLoS ONE, 5, e9022 10.1371/journal.pone.0009022 20126310 PMC2814866

[phy214359-bib-0050] Rajia, S. , Chen, H. , & Morris, M. J. (2010). Maternal overnutrition impacts offspring adiposity and brain appetite markers‐modulation by postweaning diet. Journal of Neuroendocrinology, 22, 905–914. 10.1111/j.1365-2826.2010.02005.x 20403085

[phy214359-bib-0051] Rehfeldt, C. , Lefaucheur, L. , Block, J. , Stabenow, B. , Pfuhl, R. , Otten, W. , … Kalbe, C. (2012). Limited and excess protein intake of pregnant gilts differently affects body composition and cellularity of skeletal muscle and subcutaneous adipose tissue of newborn and weanling piglets. European Journal of Nutrition, 51, 151–165. 10.1007/s00394-011-0201-8 21559991

[phy214359-bib-0052] Roever, L. , Resende, E. S. , Veloso, F. C. , Diniz, A. L. , Penha‐Silva, N. , Casella‐Filho, A. , … Chagas, A. C. (2015). Perirenal fat and association with metabolic risk factors: The uberlandia heart study. Medicine., 94, e1105 10.1097/MD.0000000000001105 26402796

[phy214359-bib-0053] Roseboom, T. , de Rooij, S. , & Painter, R. (2006). The Dutch famine and its long‐term consequences for adult health. Early Human Development, 82, 485–491. 10.1016/j.earlhumdev.2006.07.001 16876341

[phy214359-bib-0054] Roseboom, T. J. , van der Meulen, J. H. P. , Osmond, C. , Barker, D. J. P. , Ravelli, A. C. J. , Schroeder‐Tanka, J. M. , … Bleker, O. P. (2000). Coronary heart disease after prenatal exposure to the Dutch famine, 1944–45. Heart, 84, 595–598. 10.1136/heart.84.6.595 11083734 PMC1729504

[phy214359-bib-0055] Safayi, S. , Theil, P. K. , Hou, L. , Engbaek, M. , Norgaard, J. V. , Sejrsen, K. , & Nielsen, M. O. (2010). Continuous lactation effects on mammary remodeling during late gestation and lactation in dairy goats. Journal of Dairy Science, 93, 203–217. 10.3168/jds.2009-2507 20059919

[phy214359-bib-0056] Schusterova, I. , Leenen, F. , Jurko, A. , Sabol, F. , & Takacova, J. (2014). Epicardial adipose tissue and cardiometabolic risk factors in overweight and obese children and adolescents. Pediatric Obesity, 9, 63–70. 10.1111/j.2047-6310.2012.00134.x 23504985

[phy214359-bib-0057] Sharkey, D. , Symonds, M. E. , & Budge, H. (2009). Adipose tissue inflammation: Developmental ontogeny and consequences of gestational nutrient restriction in offspring. Endocrinology, 150, 3913–3920. 10.1210/en.2008-1784 19423760

[phy214359-bib-0058] Skurk, T. , Alberti‐Huber, C. , Herder, C. , & Hauner, H. (2007). Relationship between adipocyte size and adipokine expression and secretion. Journal of Clinical Endocrinology and Metabolism, 92, 1023–1033. 10.1210/jc.2006-1055 17164304

[phy214359-bib-0059] Sniderman, A. D. , Bhopal, R. , Prabhakaran, D. , Sarrafzadegan, N. , & Tchernof, A. (2007). Why might South Asians be so susceptible to central obesity and its atherogenic consequences? The adipose tissue overflow hypothesis. International Journal of Epidemiology, 36, 220–225. 10.1093/ije/dyl245 17510078

[phy214359-bib-0060] Spalding, K. L. , Arner, E. , Westermark, P. O. , Bernard, S. , Buchholz, B. A. , Bergmann, O. , … Arner, P. (2008). Dynamics of fat cell turnover in humans. Nature, 453, 783–787. 10.1038/nature06902 18454136

[phy214359-bib-0061] Spalding, K. L. , Bernard, S. , Näslund, E. , Salehpour, M. , Possnert, G. , Appelsved, L. , … Arner, P. (2017). Impact of fat mass and distribution on lipid turnover in human adipose tissue. Nature Communications, 8, 15253 10.1038/ncomms15253 PMC5457499 28534500

[phy214359-bib-0062] Suganami, T. , Nishida, J. , & Ogawa, Y. (2005). A paracrine loop between adipocytes and macrophages aggravates inflammatory changes: Role of free fatty acids and tumor necrosis factor α. Arteriosclerosis, Thrombosis, and Vascular Biology, 25, 2062–2068. 10.1161/01.ATV.0000183883.72263.13 16123319

[phy214359-bib-0063] Sun, K. , Asterholm, I. W. , Kusminski, C. M. , Bueno, A. C. , Wang, Z. V. , Pollard, J. W. , … Scherer, P. E. (2012). Dichotomous effects of VEGF‐A on adipose tissue dysfunction. Proceedings of the National Academy of Sciences USA, 109, 5874–5879. 10.1073/pnas.1200447109 PMC3326476 22451920

[phy214359-bib-0064] Symonds, M. E. , Bloor, I. , Ojha, S. , & Budge, H. (2017). The placenta, maternal diet and adipose tissue development in the newborn. Annals of Nutrition & Metabolism, 70, 232–235. 10.1159/000464301 28301844

[phy214359-bib-0065] Symonds, M. E. , Bryant, M. J. , Clarke, L. , Darby, C. J. , & Lomax, M. A. (1992). Effect of maternal cold exposure on brown adipose tissue and thermogenesis in the neonatal lamb. Journal of Physiology, 455, 487–502. 10.1113/jphysiol.1992.sp019313 1484361 PMC1175656

[phy214359-bib-0066] Symonds, M. E. , Mostyn, A. , Pearce, S. , Budge, H. , & Stephenson, T. (2003). Endocrine and nutritional regulation of fetal adipose tissue development. Journal of Endocrinology, 179, 293–299. 10.1677/joe.0.1790293 14656200

[phy214359-bib-0067] Symonds, M. E. , Phillips, I. D. , Anthony, R. V. , Owens, J. A. , & McMillen, I. C. (1998). Prolactin receptor gene expression and foetal adipose tissue. Journal of Neuroendocrinology, 10, 885–890. 10.1046/j.1365-2826.1998.00275.x 9831264

[phy214359-bib-0068] Symonds, M. E. , Pope, M. , Sharkey, D. , & Budge, H. (2012). Adipose tissue and fetal programming. Diabetologia, 55, 1597–1606. 10.1007/s00125-012-2505-5 22402988

[phy214359-bib-0069] Tanaka, T. , Yoshida, N. , Kishimoto, T. , & Akira, S. (1997). Defective adipocyte differentiation in mice lacking the C/EBPbeta and/or C/EBPdelta gene. EMBO Journal, 16, 7432–7443. 10.1093/emboj/16.24.7432 9405372 PMC1170343

[phy214359-bib-0070] Tran, T. T. , Yamamoto, Y. , Gesta, S. , & Kahn, C. R. (2008). Beneficial effects of subcutaneous fat transplantation on metabolism. Cell Metabolism, 7, 410–420. 10.1016/j.cmet.2008.04.004 18460332 PMC3204870

[phy214359-bib-0071] Umekawa, T. , Sugiyama, T. , Du, Q. , Murabayashi, N. , Zhang, L. , Kamimoto, Y. , … Ikeda, T. (2015). A maternal mouse diet with moderately high‐fat levels does not lead to maternal obesity but causes mesenteric adipose tissue dysfunction in male offspring. Journal of Nutritional Biochemistry, 26, 259–266. 10.1016/j.jnutbio.2014.10.012 25533905

[phy214359-bib-0072] Virtue, S. , & Vidal‐Puig, A. (2008). It's not how fat you are, it's what you do with it that counts. PLoS Biology, 6, e237 10.1371/journal.pbio.0060237 18816166 PMC2553843

[phy214359-bib-0073] Wallace, J. M. , Milne, J. S. , Aitken, R. P. , Redmer, D. A. , Reynolds, L. P. , Luther, J. S. , … Adam, C. L. (2015). Undernutrition and stage of gestation influence fetal adipose tissue gene expression. Journal of Molecular Endocrinology, 54, 263–275. 10.1530/JME-15-0048 25917833 PMC4449808

[phy214359-bib-0074] Weisberg, S. P. , McCann, D. , Desai, M. , Rosenbaum, M. , Leibel, R. L. , & Ferrante, A. W. Jr (2003). Obesity is associated with macrophage accumulation in adipose tissue. Journal of Clinical Investigation, 112, 1796–1808. 10.1172/JCI200319246 14679176 PMC296995

[phy214359-bib-0075] Williams, P. J. , Kurlak, L. O. , Perkins, A. C. , Budge, H. , Stephenson, T. , Keisler, D. , … Gardner, D. S. (2007). Hypertension and impaired renal function accompany juvenile obesity: The effect of prenatal diet. Kidney International, 72, 279–289. 10.1038/sj.ki.5002276 17429340 PMC2040116

[phy214359-bib-0076] Wong, K. K. L. , Wang, D. , Ko, J. K. L. , Mazumdar, J. , Le, T.‐T. , & Ghista, D. (2017). Computational medical imaging and hemodynamics framework for functional analysis and assessment of cardiovascular structures. BioMedical Engineering OnLine, 16, 35–35. 10.1186/s12938-017-0326-y 28327144 PMC5359907

[phy214359-bib-0077] Wronkowitz, N. , Romacho, T. , Sell, H. , & Eckel, J. (2014). Adipose tissue dysfunction and inflammation in cardiovascular disease. Frontiers of Hormone Research. 43, 79–92. 24943300 10.1159/000360560

[phy214359-bib-0078] Xu, H. , Barnes, G. T. , Yang, Q. , Tan, G. , Yang, D. , Chou, C. J. , … Chen, H. (2003). Chronic inflammation in fat plays a crucial role in the development of obesity‐related insulin resistance. Journal of Clinical Investigation, 112, 1821–1830. 10.1172/JCI200319451 14679177 PMC296998

[phy214359-bib-0079] Yamaguchi, Y. , Cavallero, S. , Patterson, M. , Shen, H. , Xu, J. , Kumar, S. R. , & Sucov, H. M. (2015). Adipogenesis and epicardial adipose tissue: A novel fate of the epicardium induced by mesenchymal transformation and PPARgamma activation. Proceedings of the National Academy of Sciences of the United States of America, 112, 2070–2075. 25646471 10.1073/pnas.1417232112 PMC4343131

[phy214359-bib-0080] Yan, H. , Zheng, P. , Yu, B. , Yu, J. , Mao, X. , He, J. , … Chen, D. (2017). Postnatal high‐fat diet enhances ectopic fat deposition in pigs with intrauterine growth retardation. European Journal of Nutrition, 56, 483–490. 10.1007/s00394-015-1093-9 26707995

[phy214359-bib-0081] Zuo, Y. , Qiang, L. , & Farmer, S. R. (2006). Activation of CCAAT/enhancer‐binding protein (C/EBP) alpha expression by C/EBP beta during adipogenesis requires a peroxisome proliferator‐activated receptor‐gamma‐associated repression of HDAC1 at the C/ebp alpha gene promoter. Journal of Biological Chemistry, 281, 7960–7967. 16431920 10.1074/jbc.M510682200

