# Supplementary figures and images for: Differential impacts of late gestational over–and undernutrition on adipose tissue traits and associated visceral obesity risk upon exposure to a postnatal high‐fat diet in adolescent sheep
Source: Physiol Rep. 2020 Feb 5;8(3):e14359. doi: 10.14814/phy2.14359 (PMC7002533; doi:10.14814/phy2.14359)

**HIGH-CONV**

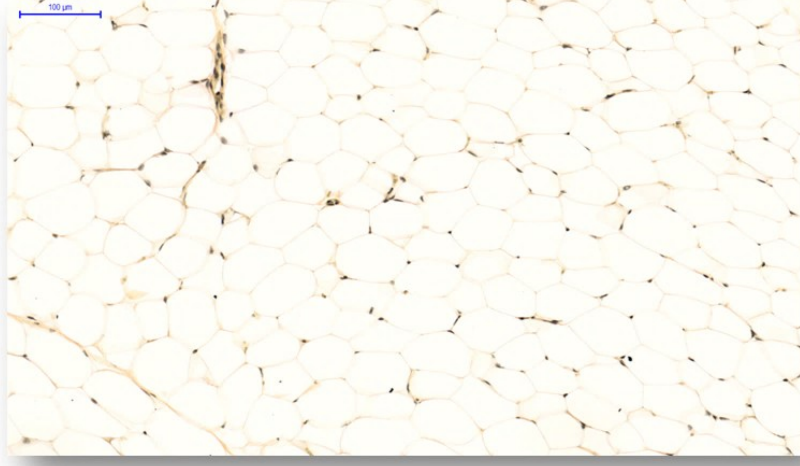

**LOW-CONV**

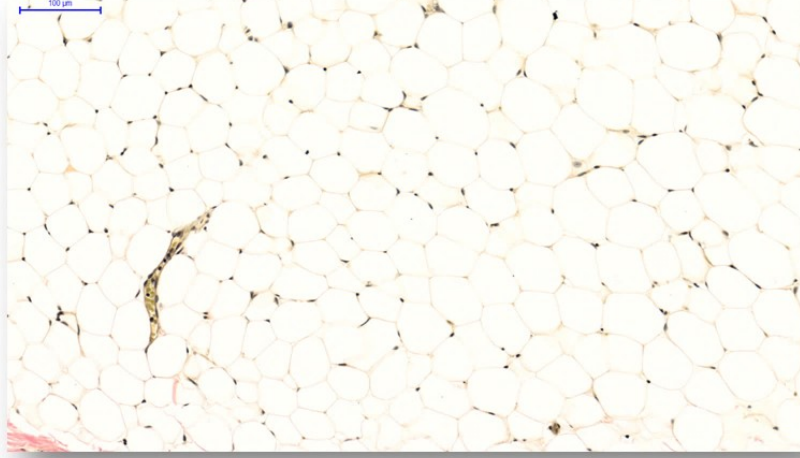

**NORM-CONV**

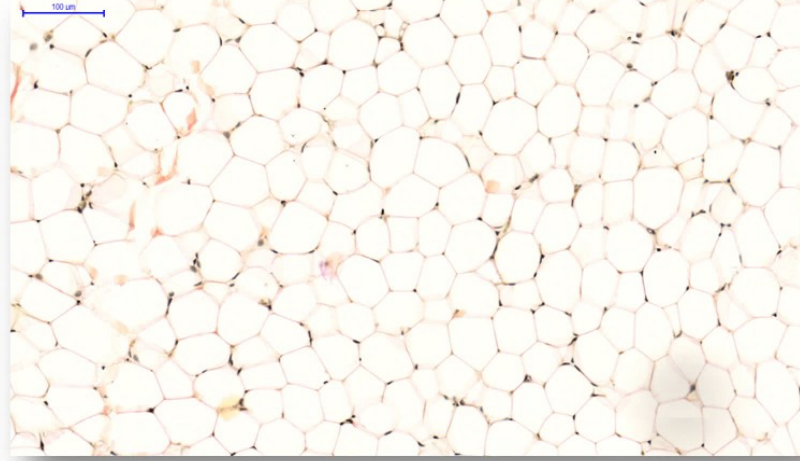

**HIGH-HCHF**

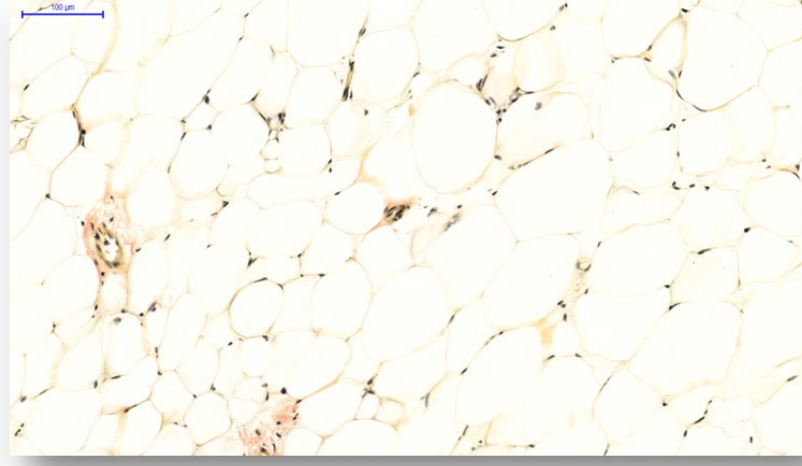

**LOW-HCHF**

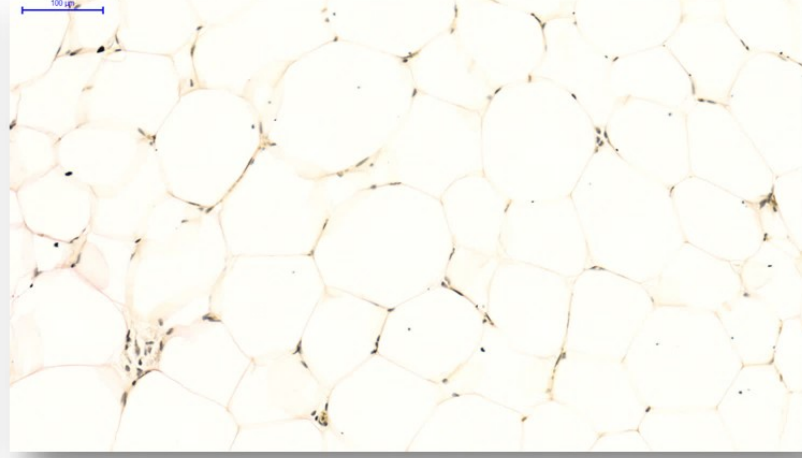

**NORM-HCHF**

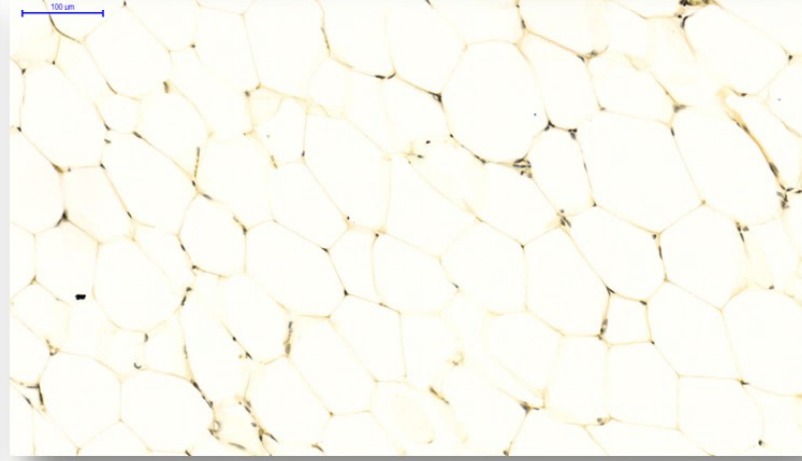

Supplement: Supplementary file 1 [file PHY2-8-e14359-s001.pdf]

**A)**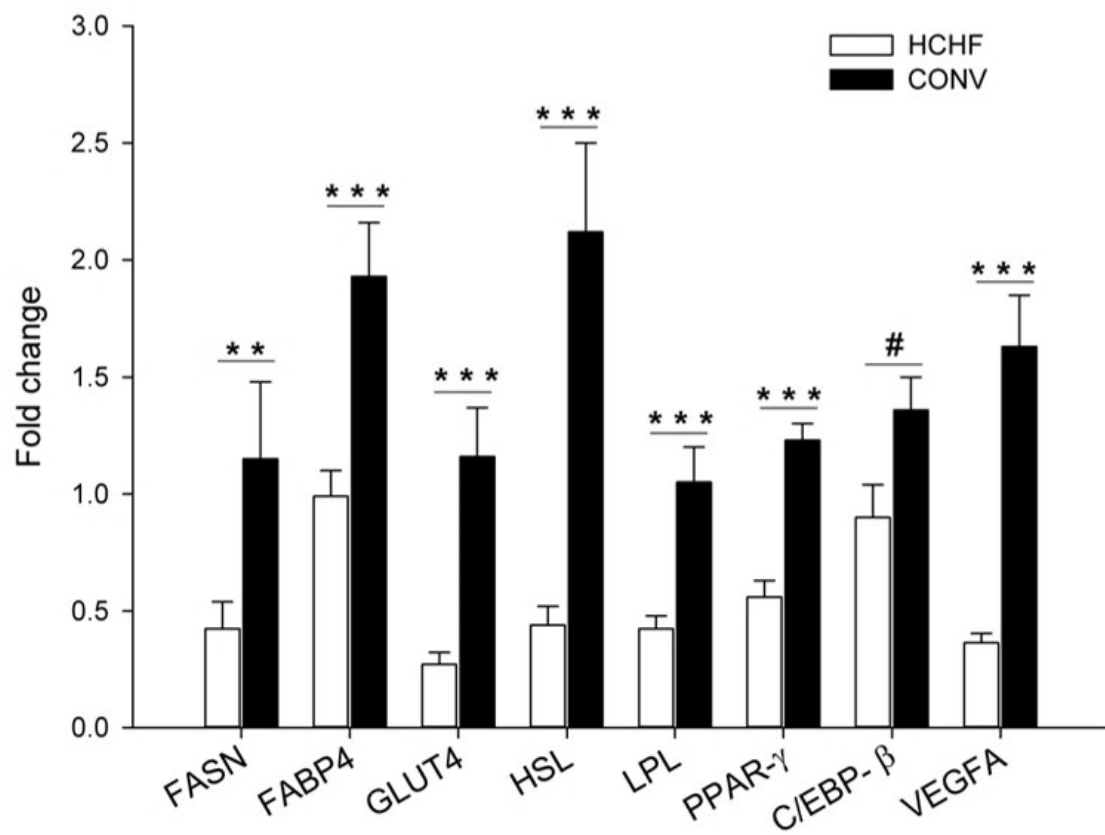**B)**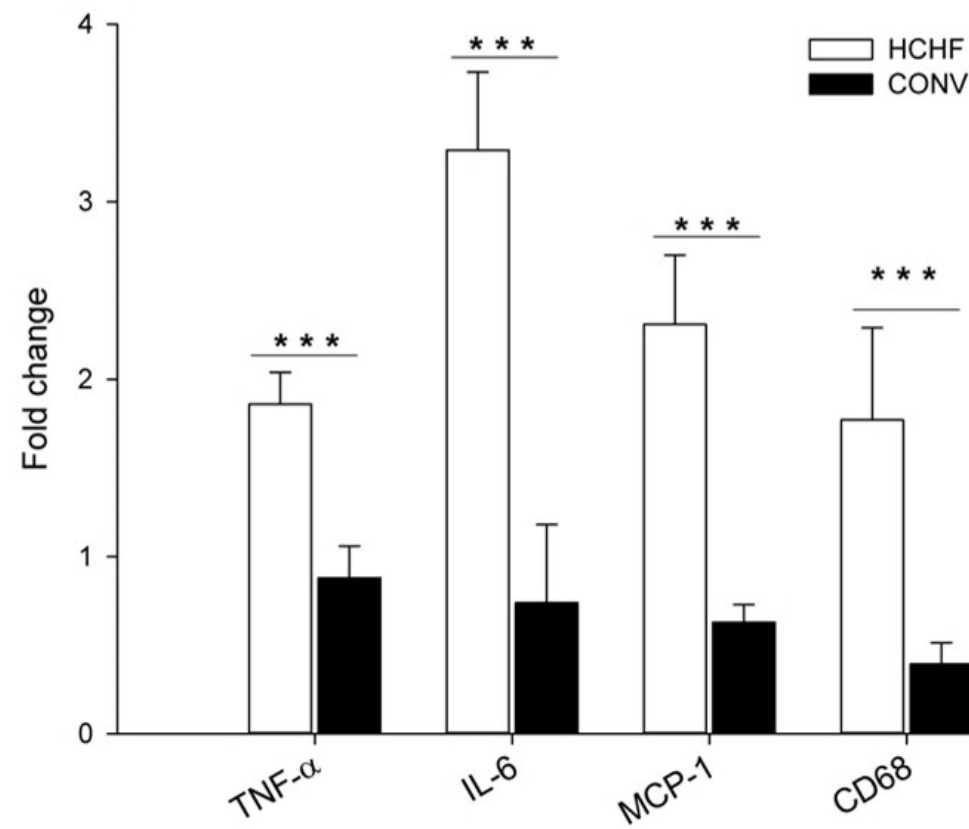

Supplement: Supplementary file 2 [file PHY2-8-e14359-s002.pdf]

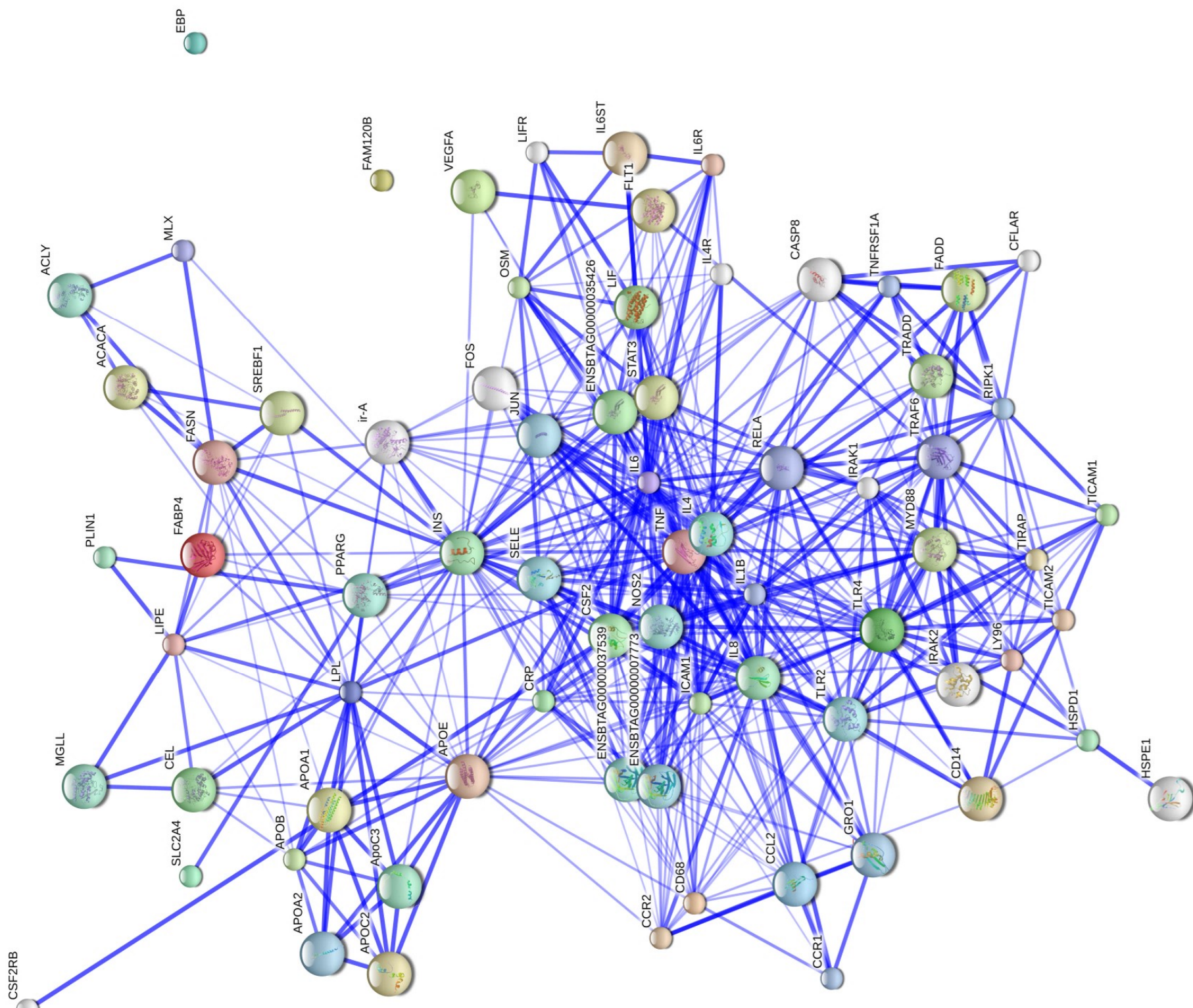

Supplement: Supplementary file 3 [file PHY2-8-e14359-s003.pdf]

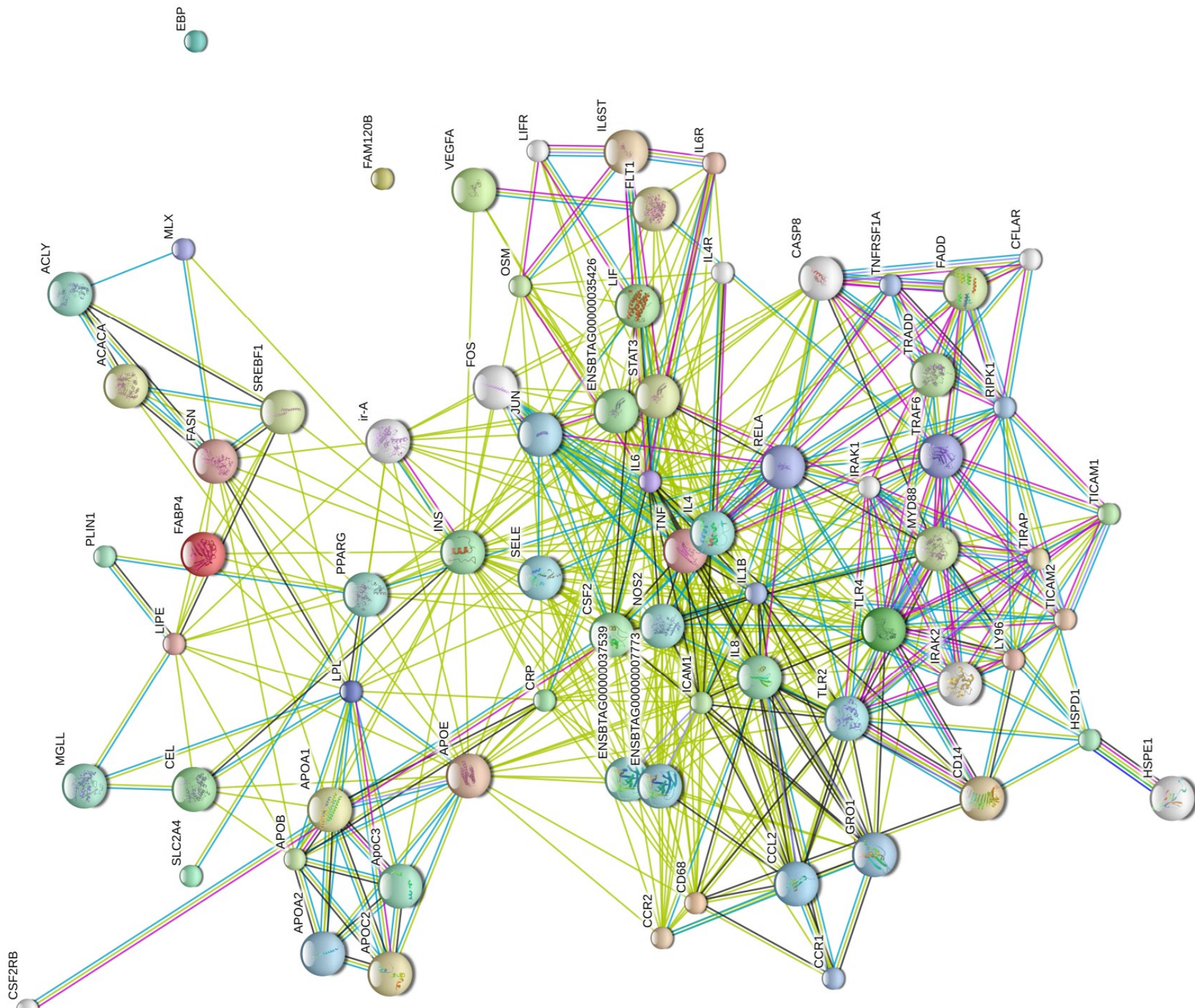

Supplement: Supplementary file 4 [file PHY2-8-e14359-s004.pdf]
